# A three-step, one-pot strategy to unsymmetrical 1,1-diarylalkenes

**DOI:** 10.1186/s13104-025-07553-0

**Published:** 2025-11-10

**Authors:** Vijayaragavan Elumalai, Hanna Bähr, Karoline Nordli, Stian R. Martinsen, Cole Funk, Jørn H. Hansen

**Affiliations:** Department of Chemistry, UiT The Arctic University of Norway, Chemical Synthesis and Analysis Group, 9037 Tromsø, Norway

**Keywords:** 1,1-Diarylalkenes, One-pot procedure, Suzuki-Miyaura coupling, Dibromination

## Abstract

**Objective:**

Develop a one-pot, three-step procedure for effective access to diverse unsymmetrical 1,1-diarylalkenes from readily accessible reagents, catalysts and substrates.

**Results:**

A three-step, one-pot procedure for the synthesis of unsymmetrical 1,1-diarylalkenes via a dibromination-elimination-Suzuki Miyaura coupling sequence has been designed and a range of reaction conditions have been screened. Although high selectivities can be achieved in some cases, the overall reaction typically proceeds in low to moderate isolated yields albeit up to 70% was observed. The reaction is compatible with a range of substituent patterns on the arylboronic acid and different styrenes.

**Supplementary Information:**

The online version contains supplementary material available at 10.1186/s13104-025-07553-0.

## Introduction

The drive towards more sustainable and green processes has enormous ramifications for synthetic chemistry. One emerging strategy to achieve increased efficiency and improved atom economy whilst reducing energy use, resources and chemical waste, is to conduct multistep reactions in single vessels (one-pot reactions) [1]. Three categories of such reactions can be discerned; (1) Cascade/tandem reactions, in which reagents are mixed in a single operation and the multiple reaction steps happen spontaneously without further intervention [2]. (2) Multicomponent reactions (MCR), in which there is also a single operation step but with three or more reactants coupling [3]. In (3), one-pot stepwise synthesis (OPSS) there are multiple operation steps with variable conditions [3, 4].

1,1-Diarylalkenes have a range of important applications in synthesis as precursors to 1,1-diarylethanes [5], in catalysis [6] and as components of bioactive substances [7]. The most common synthetic methods for these scaffolds involve Wittig-reactions and Grignard additions to aceto- or benzophenones with subsequent dehydration [8], but also other methods have emerged [9–12]. Access to more structural diversity of 1,1-diarylalkenes and increasing drive towards higher efficiency highlights the need of new synthetic methods.

In this research note, we describe our efforts to design and explore the scope of a one-pot, three-step approach to unsymmetrical 1,1-diarylalkenes involving a bromination-elimination-cross-coupling sequence, which would enable rapid access to such structures and with a high diversification potential.

## Main text

## Results and discussion

**Fig. 1:**
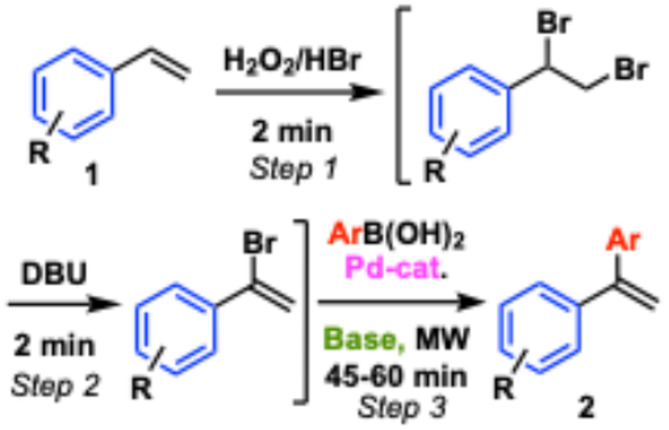
A three-step one-pot approach to unsymmetrical diarylalkenes

We have previously investigated rapid bromination chemistry using the H_2_O_2_/HBr-combination and high-yielding, selective bromination of arenes can be effected in a few minutes under optimal conditions [13, 14]. We envisioned that such conditions would rapidly dibrominate styrenes (**1**), and could undergo selective elimination of the distal bromide under similar conditions [15]. Furthermore, if the resulting 1-bromo-1-arylalkene could effectively undergo a Suzuki-Miyaura cross coupling [12], one could combine the three steps in a convenient one-pot procedure employing readily accessible starting materials, reagents and catalysts to generate unsymmetrical 1,1-diarylalkenes **2** (Fig. 1).

**Fig. 2:**
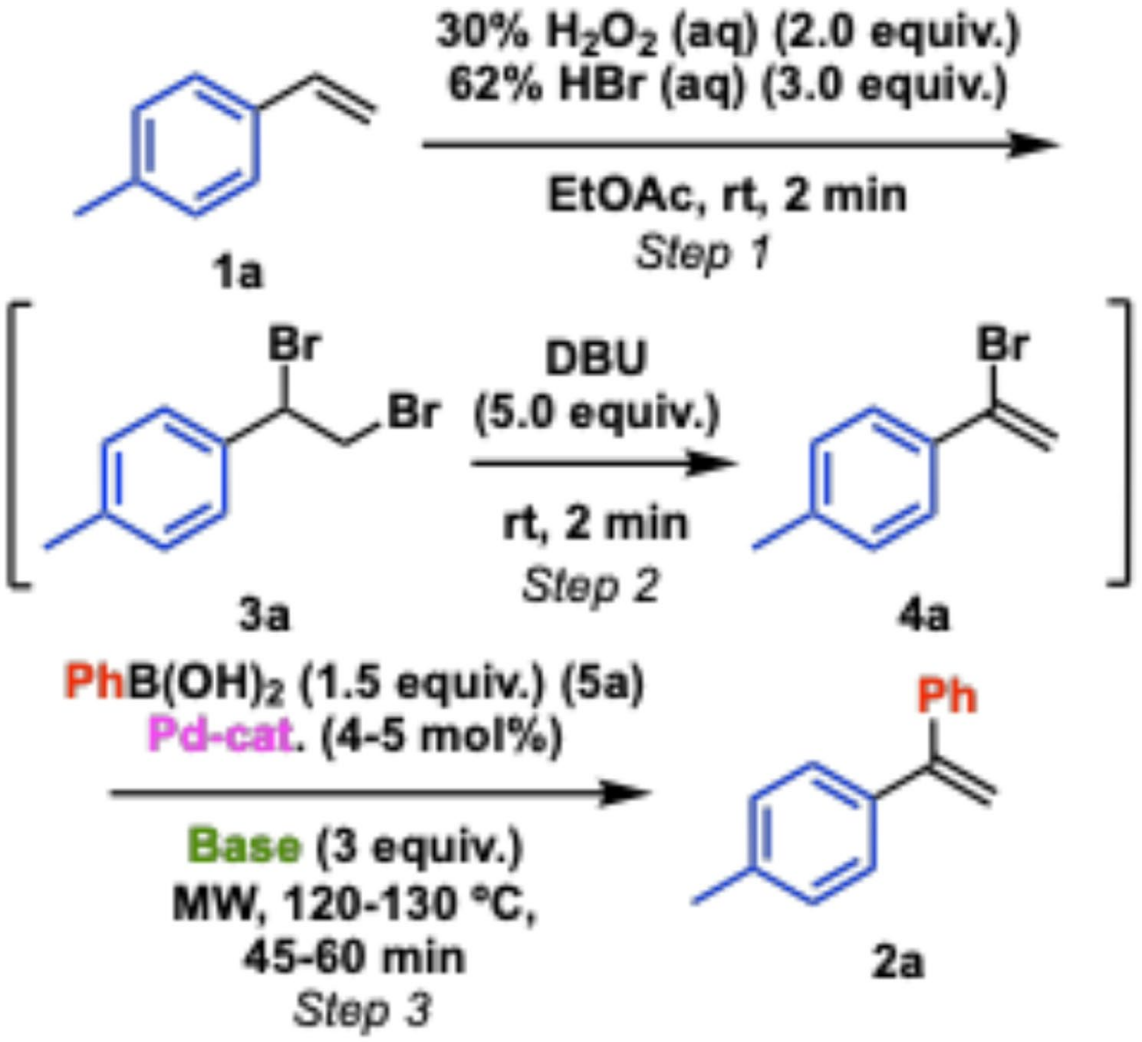
One-pot, three-step reaction conditions and intermediates

A range of conditions have been surveyed. We found that ethyl acetate was a convenient and compatible solvent for all three steps considering both performance in step 1 and solubility of reagents in further steps (Table S1). The conditions for step 1 were directly applied from our former literature procedure [13, 14], and for step 2 a detailed conditions survey is shown in the Supporting Information file (Table S2). The major side-product is the elimination product with terminal bromide (both *E* and *Z*-isomers). In Table 1 we demonstrate the influence of selected catalysts and bases on the GC-conversion of **1a** to **2a** (Fig. 2). The reactions have been monitored by taking out a reaction aliquot between each step for GC-MS analysis, and the GC-conversions to dibrominated styrene **3a** (step 1) are consistent across the entries (89–94%). Similarly, the conversions to bromoarylalkene **4a** (step 2) are consistently in the range 82–89%. The largest variation occurs in step 3 where the range is 50–90%. Surveying three common palladium catalysts at 4 mol% loading using potassium carbonate as base afforded 59–81% conversions, whereas increasing the catalyst loading to 8 mol% gave a slight increase (89%). The most promising catalyst appeared to be PdCl_2_(PPh_3_)_2_, and was therefore selected for further screening. Switching base to sodium carbonate did not offer any improvement, whereas cesium carbonate appeared to give most product (88–90% GC-conversion) and this was selected for further scope studies.

**Table 1:** Survey of step-wise GC-conversions and influence of selected catalysts and bases

Entry	Pd-cat.	Base	Rel. yield 3a (%)	Rel. yield 4a (%)	Rel. yield 2a (%)
1	PdCl_2_(PPh_3_)_2_	K_2_CO_3_	92	86	67–81
2	Pd(OAc)_2_	K_2_CO_3_	94	89	59–84
3	Pd(dba)_3_	K_2_CO_3_	91	83	63
4^a^	Pd(dba)_3_	K_2_CO_3_	91	85	89
5	PdCl_2_(PPh_3_)_2_	Na_2_CO_3_	89	82	50–79
**6**	**PdCl**_**2**_ **(PPh**_**3**_ **)**_**2**_	**Cs**_**2**_ **CO**_**3**_	**89**	**88**	**88–90**
7	PdCl_2_(PPh_3_)_2_	KO^*t*^Bu	92	85	86

GC-conversion was estimated as the product peak area percentage of the total peak areas. Entry 6 shows the conditions that gave the highest rel. yield of 2a

^a^ 8 mol% catalyst loading

**Fig. 3:**
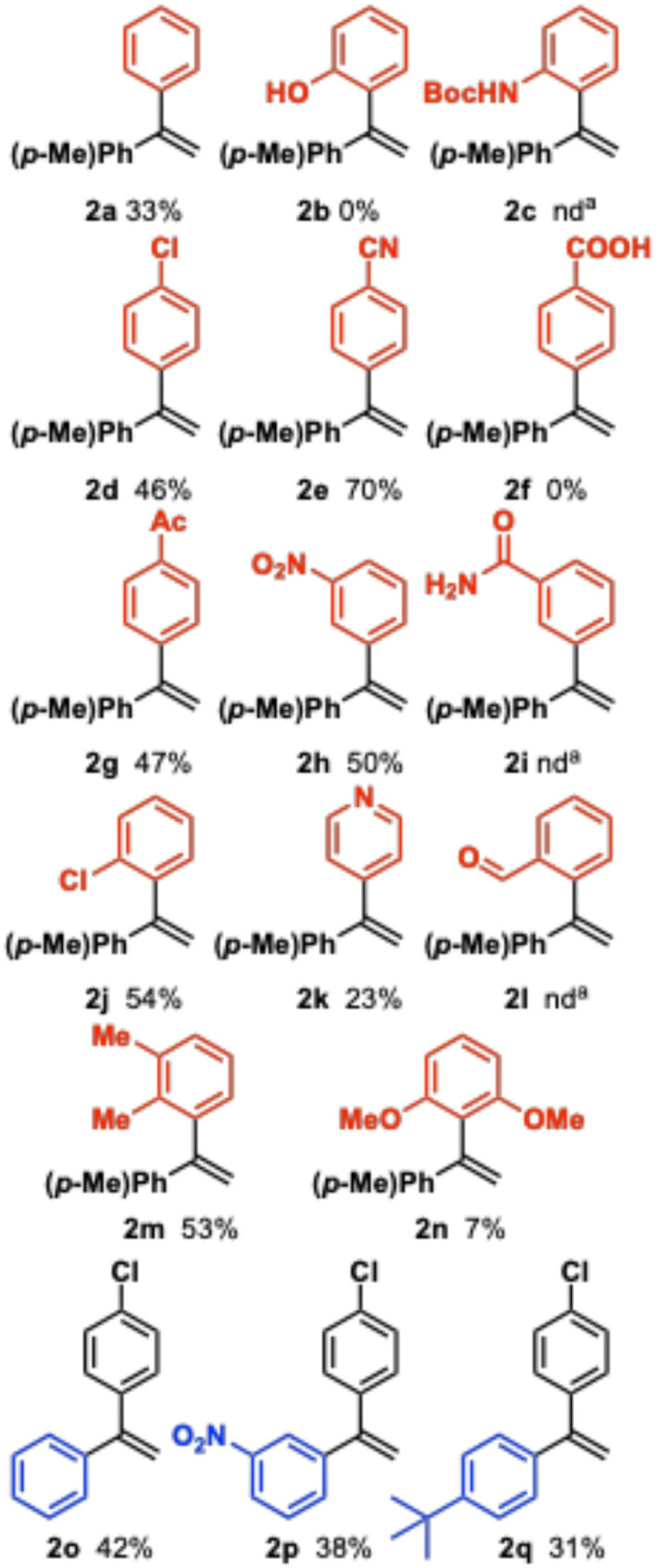
Isolated yields of products. **2a–n** are derived from various boronic acids in step 3, whereas **2o–q** represent diverse styrenes in step 1. ^a^Not determined—product trace was observed by MS but could not be isolated

With reasonably selective conditions, we next surveyed a range of arylboronic acids **5a–n** in the reaction with **1a** and **1o–q** (Fig. 3) to assess the synthetic utility of the designed one-pot process. The isolated yields are low to moderate in the range 0–54%, with a single exception for **2e** (70%). The isolated yields are lower than expected with the observed conversions in Table 1. Likely, the final cross-coupling step is underperforming in the sequence due to a complex mixture at the final stage. Moreover, the product alkenes are known to be prone to polymerization and significant product loss can occur upon isolation. The best conditions for the model system afforded 33% isolated yield of **2a**. None of the compounds containing polar H-bonding functional groups (**2b–c**,** 2f**,** 2i**, and also **2l**) were isolated under the reaction conditions, albeit traces of **2c**, **2i** and **2l** were detected by MS. For the remaining *p*-series with chloro- (**2d**), cyano- (**2e**) and acetyl- (**2g**) substituents, yields of 45–70% were observed. The *meta-*nitro substituted product **2h** was also isolated in 50% yield. The *ortho*-chloro-substituted **2j** was formed in 54% yield and the 2,3-dimethyl substituted system **2m** in 53% yield, and shows that the method tolerates both *o-* and *m-*substitution. For comparison, the more crowded *o*-dimethoxy-system **2n** gave only 7% yield. The formation of 23% of 4-pyridyl-substituted **2k** demonstrates some compatibility with heterocycles. Finally, three different styrenes **1o–q** were tested in the reaction using the *p*-chloro-substituted boronic acid yielding 31–42% of **2o–q**, which is roughly as expected in comparison to **2d**. The results demonstrate the feasibility of the reaction with different styrenes and boronic acids, albeit in overall low to moderate yields.

In summary, we have designed and investigated a range of reaction conditions for a rapid three-step, one-pot approach to unsymmetrical 1,1-diarylalkenes. Although high conversions can be achieved for all the steps in the sequence, the last step has a large conversion variance and the final products can be isolated mostly in low to moderate yields of 0–54%. The reaction conditions for specific substrate combinations may be susceptible to further optimization for each substrate demonstrated by the 70% isolated yield of **2e**. The method may be valuable for library generation or late-stage modification where operational simplicity is crucial whilst moderate yields are acceptable.

## Methods

Unless otherwise noted, purchased chemicals were used as received without further purification. Thin layer chromatography was carried out using TLC Silica Gel 60 F254 (Merck) and visualized by short-wavelength ultraviolet light or by treatment with an appropriate stain. Microwave reactions were conducted in a Monowave 300 by Anton Paar. Flash chromatography was carried out on silica gel 60 (230–400 mesh). Autoflash chromatography (normal and reversed phase) was conducted on CombiFlash EZ prep system. Normal-phase chromatography was performed on RediSep^®^Rf High Performance Gold columns in the appropriate size with the sample preloaded on a precolumn containing celite. Prep-HPLC column chromatography was conducted on a YMC-Actus Triart C18 Semi-preparative HPLC column, 12 nm, S-5 μm 20 × 150 mm 5 μm with a YMC-Triart C18, semi-preparative Guard Cartridge incl. Sealing, 12 nm, S-5 μm, 10 × 20. The sample was liquid loaded pure or mixed with a suitable solvent. Solvent systems are reported as follows: (solventA: solventB [the percentage of solvent]), when the autoflash system was used. High-resolution mass spectra HRMS(ESI) were recorded from methanol solutions on a LTQ Orbitrap XL (Thermo Scientific) in either positive or negative electrospray ionization (ESI) mode. NMR spectra were obtained on a 400 MHz Bruker Avance III HD at 20 °C. GC-MS analysis was performed on a TRACE GC ULTRA, ITQ 1100 instrument with a SUPELCO analytical SLB™-5ms Fused Silica Capillary Column 30 m × 0.2 μm film thickness.

## Limitations

The overall chemical yields were variable and mostly low to moderate. A major problem with the three-step sequence is a wide range of yield outcomes in the final cross-coupling step, but also the formation of other regioisomeric alkenes in step 2 and the formation of other side-products in step 1 could contribute to these results. The problems with step 3 may be alleviated by increasing the catalyst loading. Furthermore, product loss during final work-up and isolation due to instability may be a contributing factor to the observed low to moderate yields.

## Supplementary Information

Below is the link to the electronic supplementary material.

Supplementary Material 1.

## Data Availability

NMR-data for all final products can be found in a separate supplementary file and is also available from the corresponding authors upon request. No crystallographic or macromolecular structure data has been generated.

## Abbreviations

MCR: Multicomponent reactions
OPSS: One-pot stepwise synthesis
Me: Methyl
DBU: 1,8-Diazabicyclo[5.4.0]undec-7-ene
MW: Microwave
GC–MS: Gas chromatography–mass spectrometry
Ph: Phenyl
Ar: Aryl
Ac: Acetyl
Et: Ethyl
HRMS: High-resolution mass spectrometry
rt: Room temperature
Boc: *tert*-butyloxycarbonyl
