# Supplementary material for: A three-step, one-pot strategy to unsymmetrical 1,1-diarylalkenes
Source: BMC Res Notes. 2025 Nov 10;18:474. doi: 10.1186/s13104-025-07553-0 (PMC12604287; doi:10.1186/s13104-025-07553-0)
Supplement: Supplementary file 1 — Supplementary Material 1. [file 13104_2025_7553_MOESM1_ESM.pdf]

SUPPORTING INFORMATION FOR:

A three-step, one-pot strategy to unsymmetrical 1,1-diarylalkenes

*Vijayaragavan Elumalai, Hanna Bähr, Karoline Nordli, Stian R. Martinsen, Cole Funk and Jørn H. Hansen**

Department of Chemistry, UiT The Arctic University of Norway, Chemical Synthesis and Analysis Group,

N-9037 Tromsø, Norway

** Corresponding author e-mail: jorn.h.hansen@uit.no*

Table S1: Survey of solvent influence on the conversion to 2 (step 1).

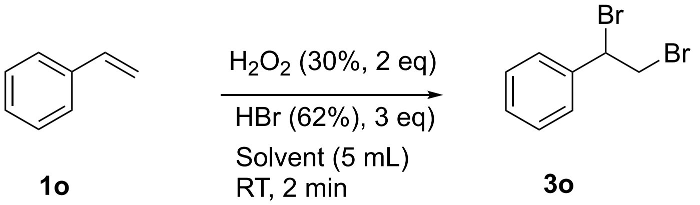

#	Solvent	Conversion to **3o** (%)[a,b]
1	MeOH	18
2	EtOH	15
3	H_2O	0
4	EtOAc	**99**
5	AcCN	**94**
6	Acetone	**50**
7	THF	**86**
8	DCM	**93**
9	Toluene	**99**

(a) **Reaction Procedure**: To a stirred solution of styrene **1o** (2.92 mmol) in a specified solvent, was added H_2O_2 (30%, 0.6 mL, 2 equiv) and HBr (62%, 0.66 mL, 3 equiv.) The reaction mixture was stirred for 2 min. at 0 ^oC. After the reaction time, water (10 mL) was added and diluted with ethylacetate (25 mL) , washed with $Na_2S_2O_3$ (10%, 10 mL). The organic layer was dried over Na_2SO_4 and the solvent was evaporated to obtain the desired dibromo compound.
(b) GC-conversion.

Table S2: Survey of base, reaction time and temperature on GC-MS conversion in step 2.

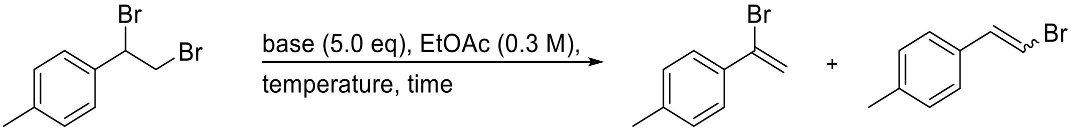

3a **4a** 1,2-**4a**

entry	Base	Time [min]	Temperature [°C]	Conversion of 3a [%][a]	Rel. yield of 4a [%][b]	Rel. yield of 1,2-4a [%][b]
**1**	K_2CO_3	10	100 (µw)	76	67	9
**2**	Na_2CO_3	10	100 (µw)	50	32	18
**3**	Et_3N	10	100 (µw)	74	30	44
**4**	DIPEA	10	100 (µw)	6	4	1
**5**	pyridine	10	100 (µw)	83	68	15
**6**	NaOH	10	100 (µw)	46	20	27
**7**	piperidine	10	100 (µw)	92	82	10
**8**	DMAP	10	100 (µw)	82	81	1
**9**	DBU	10	100 (µw)	>99	97	3
**10**	aniline	10	100 (µw)	17	15	2
**11**	DBU	5	100 (µw)	99	95	4
**12**	DBU	2	100 (µw)	99	92	7
**13**	DBU	10	80 (µw)	>99	93	7
**14**	DBU	2	50 (µw)	>99	93	7
**15**	DBU	5	rt	98	95	3
**16**	DBU	2	rt	99	91	7

[a]Conversion based on GC-MS peak areas. [b]by GC-MS.

General experimental details

Unless otherwise noted, purchased chemicals were used as received without further purification. Thin layer chromatography was carried out using TLC Silica Gel 60 F254 (Merck) and visualized by short-wavelength ultraviolet light or by treatment with an appropriate stain. Microwave reactions were conducted in a Monowave 300 by Anton Paar. Flash chromatography was carried out on silica gel 60 (230-400 mesh). Autoflash chromatography (normal and reversed phase) was conducted on CombiFlash EZ prep system. Normal-phase chromatography was performed on RediSep®Rf High Performance Gold columns in the appropriate size with the sample preloaded on a precolumn containing celite. Prep-HPLC column chromatography was conducted on a YMC-Actus Triart C18 Semi-preparative HPLC column, 12 nm, S-5 µm 20x150mm 5 µm with a YMC-Triart C18, semi-preparative Guard Cartridge incl. Sealing, 12 nm, S-5 µm, 10 x 20. The sample was liquid loaded pure or mixed with a suitable solvent. Solvent systems are reported as follows: (solventA:solventB [the percentage of solvent]), when the autoflash system was used. High-resolution mass spectra HRMS(ESI) were recorded from methanol solutions on a LTQ Orbitrap XL (Thermo Scientific) in either positive or negative electrospray ionization (ESI) mode. NMR spectra were obtained on a 400 MHz Bruker Avance III HD at 20 °C. The 1,1-diarylalkenes were confirmed to have the alkylidene protons attached to the same carbon by HSQC-experiments. GC-MS analysis was performed on a TRACE GC ULTRA, ITQ 1100 instrument with a SUPELCO analytical SLB™-5ms Fused Silica Capillary Column 30m x 0.2µm film thickness.

General procedure for one-pot reaction

The styrene (1.00 mmol, 1.00 eq) was mixed with EtOAc (3.0 mL) in a microwave vial containing a stir bar. 62% $HBr_{(aq)}$ (0.20 mL, 2.00 mmol, 2.00 eq) and 30% $H_2O_{2(aq)}$ (0.20 mL, 2.00 mmol, 2.00 eq) were added to the stirred solution. The reaction mixture was stirred for 2 min at ambient temperature. Then, DBU (0.75 mL, 5.00 mmol, 5.00 eq) was added and the mixture was stirred for 2 min at ambient temperature. Boronic acid (1.50 mmol, 1.50 eq), Cs_2CO_3 (978 mg, 3.00 mmol, 3.00 eq) and $Pd(Phh_3)_2Cl_2$ (4-5 mol%) were added. The vial was sealed and flushed with argon. The solution was sonicated for 10 s and then heated in the microwave to 120-130 ºC for 45-60 min. EtOAc was added and the organic layer was washed with H_2O and brine. The combined aqueous layers were extracted with EtOAc (2X). The combined organic layers were dried over Na_2SO_4, filtered and concentrated in *vacuo*. The crude product was purified by column chromatography on silica gel.

Characterization data

2a

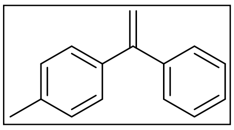

Styrene: 4-methylstyrene; boronic acid: phenylboronic acid. The crude product was purified by column chromatography (*n*-pentane). The product (64.5 mg, 0.33 mmol) was isolated, containing minor amounts of impurities, as clear crystals in 33% yield. R_f (*n*-heptane:EtOAc 3:1) = 0.78. ^{1}H-NMR (400 MHz, $CDCl_3$) δ 7.44 – 7.34 (m, 5H), 7.32 – 7.27 (m, 2H), 7.19 (d, *J* = 7.9 Hz, 2H), 5.48 (d, *J* = 1.2 Hz, 2H), 2.42 (s, 3H). ^{13}C-NMR (101 MHz, $CDCl_3$) δ 150.0, 141.8, 138.8, 137.6, 129.0, 128.9, 128.4, 128.3, 128.3, 127.8, 127.4, 127.3, 113.8, 21.3. HRMS(ESI): m/z calc. for $C_{15}H_{15}[M+H]^{+}$: 195.1168; found: 195.1172. The data is consistent with literature values (Tang et al. 2016, Xia et al. 2023).

2c

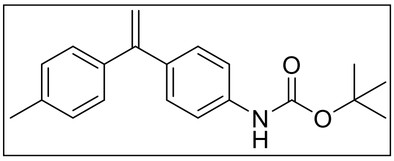

Styrene: 4-methylstyrene; boronic acid derivative: (4-((*tert*-butoxycarbonyl)amino)phenyl)boronic acid. The product could not be isolated. HRMS(ESI): m/z calc. for $C_{20}H_{24}NO_2[M+H]^{+}$: 310.1802; found: 310.1799.

2d

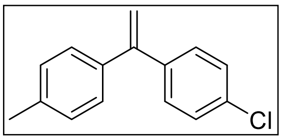

Styrene: 4-methylstyrene; boronic acid derivative: 4-chlorophenylboronic acid. The crude product was purified by column chromatography (*n*-pentane). The product (102.6 mg, 0.45 mmol) was isolated, containing minor amounts of impurities, as a clear oil in 45% yield. R_f (*n*-heptane) = 0.50. ^{1}H-NMR (400 MHz, $CDCl_3$) δ 7.33 – 7.27 (m, 4H), 7.24 – 7.14 (m, 4H), 5.43 (d, *J* = 1.2 Hz, 2H), 2.39 (s, 3H). ^{13}C-NMR (101 MHz, $CDCl_3$) δ 149.0, 140.3, 138.3, 137.9, 133.6, 129.7, 129.2, 129.1 (2C), 128.4 (2C), 128.2 (2C), 114.2, 21.3. HRMS(ESI): m/z calc. for $C_{15}H_{14}Cl[M+H]^{+}$: 229.0779; found: 229.0776. The data is consistent with literature values (Tang et al. 2016).

2e

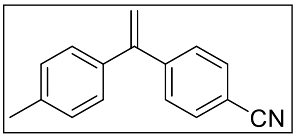

Styrene: 4-methylstyrene; boronic acid derivative: 4-cyanophenylboronic acid. The crude product was purified by column chromatography (10-20% ethyl acetatet/pentane). The resulting product was isolated as an orange solid (224 mg, 70%). R_f (EtOAc/pentane 1:9) = 0.66. ^{1}H NMR (400 MHz, $CDCl_3$): δ 7.67–7.61 (m, 2H), 7.50–7.44 (m, 2H), 7.21 (s, 4H), 5.60 (d, J = 1.0 Hz, 1H), 5.53 (d, J = 0.9 Hz, 1H), 2.42 (s, 3H). ^{13}C NMR (101 MHz, $CDCl_3$): δ 148.6, 146.3, 138.2, 137.4, 132.1, 129.2, 128.9, 128.1, 118.9, 116.1, 111.3, 21.2. HRMS (ESI): m/z calcd. for $C_{14}H_{14}N_2Na^{+}$ $[M+Na]^{+}$: 242.0940; found: 242.0940.

2g

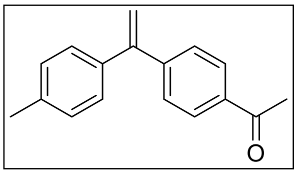

Styrene: 4-methylstyrene; boronic acid derivative: 4-acetylphenylboronic acid. The product was isolated by dry loading with flash column chromatography (silica, EtOAc/pentane 1:9) resulting in the product as a yellow solid (162 mg, 47%). R_f (pentane/EtOAc 9:1) = 0.69. MS (EI): m/z 178 (42%), 221 (100), 222 (19), 236 (65 $[M]^+$). ^{1}H NMR (400 MHz, $CDCl_3$): δ 7.85–7.79 (m, 2H), 7.38–7.28 (m, 2H), 7.13–7.03 (m, 4H), 5.43 (d, J = 1.1 Hz, 1H), 5.39 (d, J = 1.1 Hz, 1H), 2.51 (s, 3H), 2.28 (s, 3H). ^{13}C NMR (101 MHz, $CDCl_3$): δ 197.7, 149.1, 146.5, 137.9, 137.9, 136.3, 129.1, 128.5, 128.3, 128.1, 115.4, 26.7, 21.2. HRMS (ESI): m/z calcd. for $C_{14}H_{14}O_2Na^+$ $[M+Na]^+$: 259.1177; found: 259.1093. The data is consistent with literature values (Gao et al. 2024).

2h

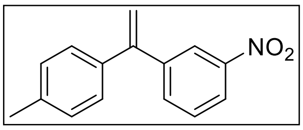

Styrene: 4-methylstyrene; boronic acid derivative: 3-nitrophenylboronic acid. The crude product was purified by column chromatography on silica gel (*n*-heptane then *n*-heptane:EtOAc 5:1). The product (120.0 mg, 0.50 mmol) was isolated, containing minor amounts of impurities, as an orange oil in 50% yield. R_f (*n*-heptane:EtOAc 3:1) = 0.67. ^{1}H-NMR (400 MHz, $CDCl_3$) δ 8.29 (t, J = 2.0 Hz, 1H), 8.24 (ddd, J = 8.1, 2.3, 1.1 Hz, 1H), 7.73 (dt, J = 7.9, 1.4 Hz, 1H), 7.56 (t, J = 7.9 Hz, 1H), 7.35 – 7.13 (m, 4H), 5.64 (d, J = 0.8 Hz, 1H), 5.58 (d, J = 0.8 Hz, 1H), 2.46 (s, 3H). ^{13}C-NMR (101 MHz, $CDCl_3$) δ 148.4, 148.1, 143.6, 138.4, 137.4, 134.4, 129.3, 129.2, 128.1, 123.1, 122.6, 115.9, 21.3. HRMS(ESI): m/z calc. for $C_{15}H_{14}NO_2[M+H]^+$: 240.1019; found: 240.1017. IR (ν_{max}/cm^{-1}, neat): 2918, 1661, 1606, 1524, 1346, 904, 810, 706. The data is consistent with literature values (Tang et al. 2016).

2i

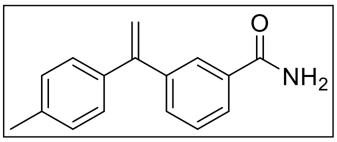

Styrene: 4-methylstyrene; boronic acid derivative: 2-aminocarbonylphenylboronic acid. The product could not be isolated. HRMS(ESI): m/z calc. for $C_{16}H_{16}NO[M+H]^+$: 238.1226; found: 238.1226.

2j

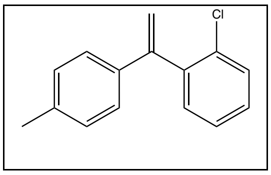

The crude product was isolated by silica flash column chromatography (pentane) and was isolated as a yellow liquid (180 mg, 54%). R_f (pentane) = 0.39. MS (EI): m/z 178 (100%), 179 (23.55), 193 (96.34), 194 (15.13), 228 (71.39), 230 (23.09). ^{1}H NMR (400 MHz, $CDCl_3$): δ 7.50–7.47 (m, 1H), 7.42–7.39 (m, 1H), 7.38–7.34 (m, 2H), 7.27 (d, J = 8.2 Hz, 2H), 7.20 (d, J = 8.2 Hz, 2H), 5.90 (d, J = 1.1 Hz, 1H), 5.32 (d, J = 1.1 Hz, 1H), 2.43 (s, 3H). ^{13}C NMR (101 MHz, $CDCl_3$): δ 147.4, 141.0, 137.7, 137.0, 133.3, 131.7, 129.7, 129.2, 128.9, 126.8, 126.4, 115.4, 21.3. The data is consistent with literature values (Chen et al. 2016).

2k

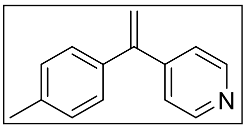

Styrene: 4-methylstyrene; boronic acid derivative: pyridine-4-boronic acid. The crude product was purified by column chromatography (*n*-heptane -> EtOAc). The product (45.0 mg, 0.23 mmol) was isolated, containing minor amounts of impurities, as a yellow oil in 23% yield. R_f (*n*-heptane:EtOAc 2:1) = 0.32 ^{1}H-NMR (400 MHz, $CDCl_3$) δ 8.66 – 8.56 (m, 2H), 7.33 – 7.21 (m, 2H), 7.27 – 7.15 (m, 4H), 5.60 (d, *J* = 0.9 Hz, 1H), 5.58 (d, *J* = 0.9 Hz, 1H), 2.40 (s, 3H). ^{13}C-NMR (101 MHz, $CDCl_3$) δ 150.0, 149.2, 147.9, 138.3, 137.0, 129.2, 128.1, 123.0, 116.4, 21.3. HRMS(ESI): m/z calc. for $C_{14}H_{14}N[M+H]^+$: 196.1121; found 196.1120. The data is consistent with the literature (Maekawa et al. 2015).

2l

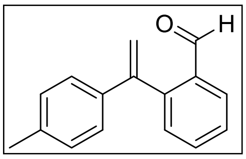

Styrene: 4-methylstyrene; boronic acid derivative: 2-formylphenylboronic acid. The product could not be isolated. HRMS(ESI): m/z calc. for $C_{17}H_{15}O[M+H]^+$: 223.1117; found: 223.1150.

2m

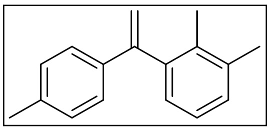

Styrene: 4-methylstyrene; boronic acid derivative: 2,3-methylboronic acid. The crude product was purified by column chromatography (*n*-pentane). The product (117.0 mg, 0.53 mmol) was isolated, containing minor amounts of impurities, as a clear oil in 53% yield. R_f (*n*-heptane) = 0.38. ^{1}H-NMR (400 MHz, $CDCl_3$) δ 7.24 – 7.06 (m, 7H), 5.80 (s, 1H), 5.17 (s, 1H), 2.37 (s, 3H), 2.33 (s, 3H), 2.04 (s, 3H). ^{13}C-NMR (101 MHz, $CDCl_3$) δ 149.8, 142.1, 138.1, 137.5, 137.1, 134.8, 129.2 (2C), 128.0, 126.5, 125.4, 113.8, 21.3, 20.6, 16.9. HRMS(ESI): m/z calc. for $C_{17}H_{19}[M+H]^+$: 223.1481; found: 223.1479. IR (ν_{max}/cm^{-1}, neat): 2919, 1720, 166, 1604, 1458, 1276, 1175, 943, 748.

2n

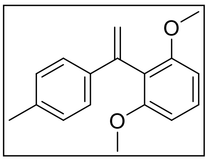

Styrene: 4-methylstyrene; boronic acid derivative: 2,6-dimethoxyphenylboronic acid. The crude product was purified by column chromatography (*n*-pentane then *n*-pentane:EtOAc 100:1). The product (17.0 mg, 0.07 mmol) was isolated, containing minor amounts of impurities, as a yellow oil (similar to reference) in 7% yield. R_f (*n*-heptane:EtOAc 3:1) = 0.62. ^{1}H-NMR (400 MHz, $CDCl_3$) δ 7.24 – 7.03 (m, 3H), 7.02 – 6.93 (m, 2H), 6.55 (d, *J* = 8.4 Hz, 2H), 5.86 (d, *J* = 1.4 Hz, 1H), 5.07 (d, *J* = 1.4 Hz, 1H), 3.63 (s, 6H), 2.23 (s, 3H). ^{13}C-NMR (101 MHz, $CDCl_3$) δ 158.2, 141.2, 137.5, 137.0, 128.9, 128.7, 125.8, 119.9, 115.0, 104.3, 56.2, 21.3. HRMS(ESI): m/z calc. for $C_{17}H_{19}O_2[M+H]^+$: 255.1380; found: 255.1377. IR (ν_{max}/cm^{-1}, neat): 2934, 1588, 1470, 1432, 1248, 1107, 787. The data is consistent with literature values (Jenthra et al. 2023).

2o

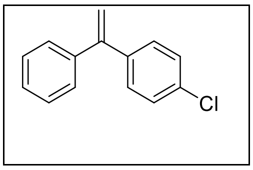

Prepared according to the general procedure. The crude product was purified by silica gel column chromatography (pentane) to obtain the desired product as a clear oil (90 mg, 42 %). ^{1}H NMR (400 MHz, $CDCl_3$) δ 7.38 – 7.29 (m, 9H), 5.48 (dd, *J* = 7.2, 1.1 Hz, 2H). ^{13}C NMR 149.1, 141.1, 140.1, 133.7, 129.7, 128.5, 128.4, 128.3, 128.1, 114.8. The data is consistent with literature (Isbrandt et al. 2024).

2p

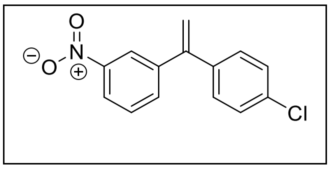

Prepared according to the general procedure. The crude product was purified by silica gel column chromatography (pentane:diethyl ether 100:0 → 100:5) to obtain the desired product as a yellow oil (99 mg, 38 %). ^{1}H NMR (400 MHz, $CDCl_3$) δ 8.23 – 8.14 (m, 2H), 7.63 (dt, *J* = 7.7, 1.4 Hz, 1H), 7.54 – 7.49 (m, 1H), 7.36 – 7.31 (m, 2H), 7.26 – 7.21 (m, 2H), 5.59 (d, *J* = 7.0 Hz, 2H). ^{13}C NMR (101 MHz, $CDCl_3$) δ 148.5, 147.2, 142.9, 138.7, 134.5, 134.2, 129.5, 129.4, 128.9, 123.1, 122.9, 117.1. The data is consistent with literature (Duong et al. 2019)

2q

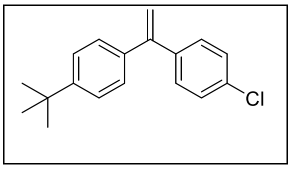

Prepared according to the general procedure. The crude product was purified by silica gel column chromatography (pentane) to obtain the desired product as a colourless oil (84 mg, 31 %). ^{1}H NMR (400 MHz, $CDCl_3$) δ 7.43 – 7.36 (m, 2H), 7.35 – 7.30 (m, 4H), 7.29 – 7.25 (m, 2H), 5.45 (dt, *J* = 26.6, 1.3 Hz, 2H), 1.37 (s, 9H). ^{13}C NMR (101 MHz, $CDCl_3$) δ 151.1, 148.9, 140.3, 138.1, 133.6, 129.8, 128.4, 128.0, 125.3, 114.3, 34.7, 31.5. The data is consistent with literature (Qin et al. 2013).

2a

^{1}H-NMR

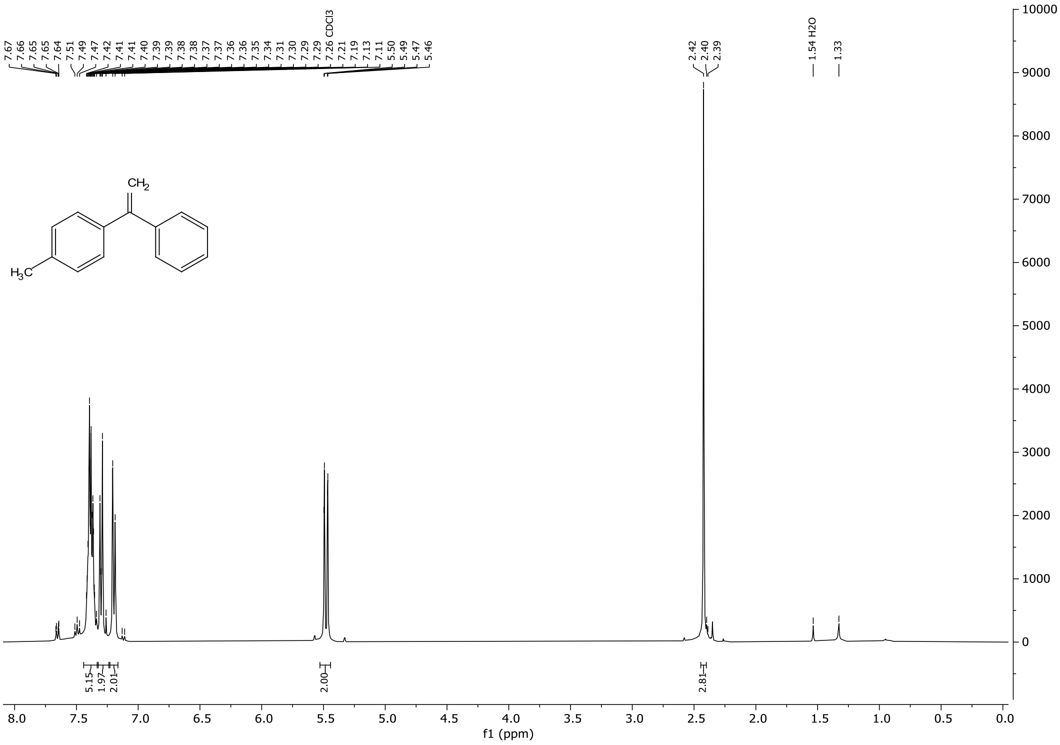

^{13}C-NMR

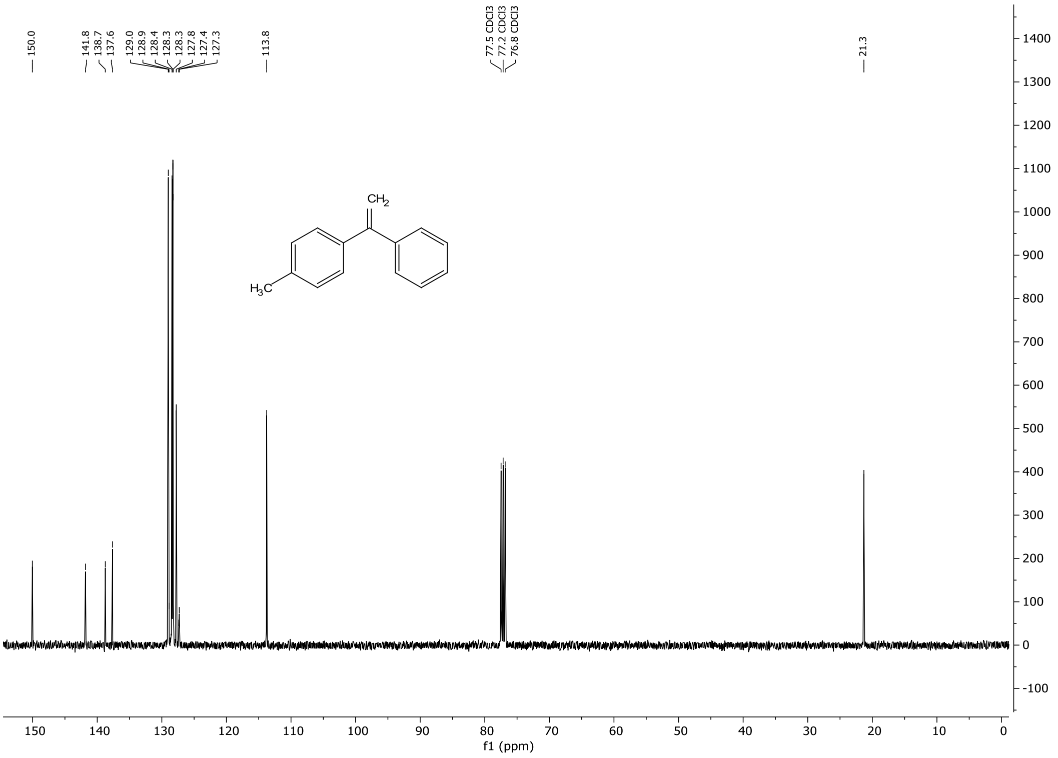

2d

^{1}H-NMR

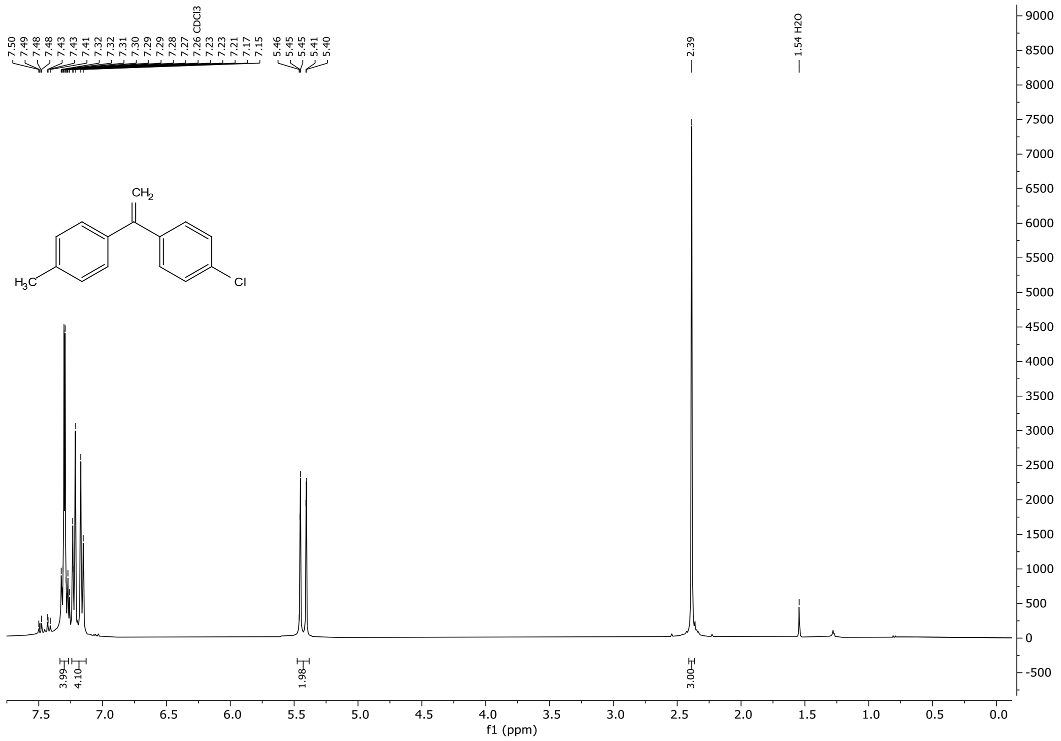

^{13}C-NMR

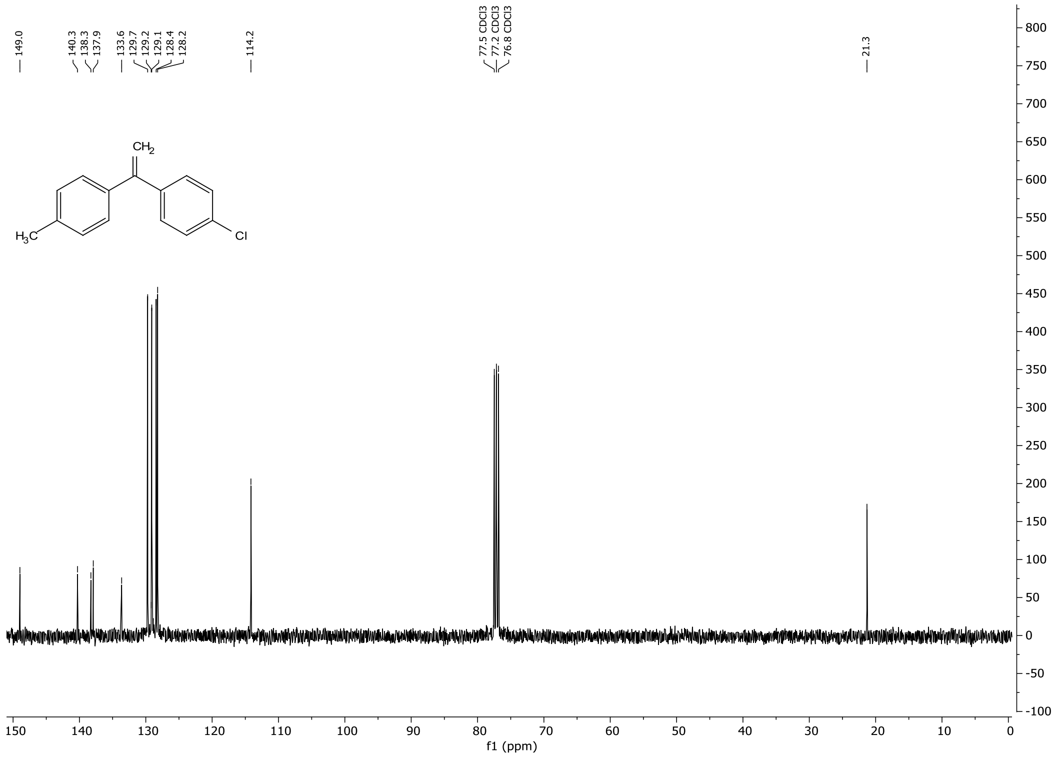

2e

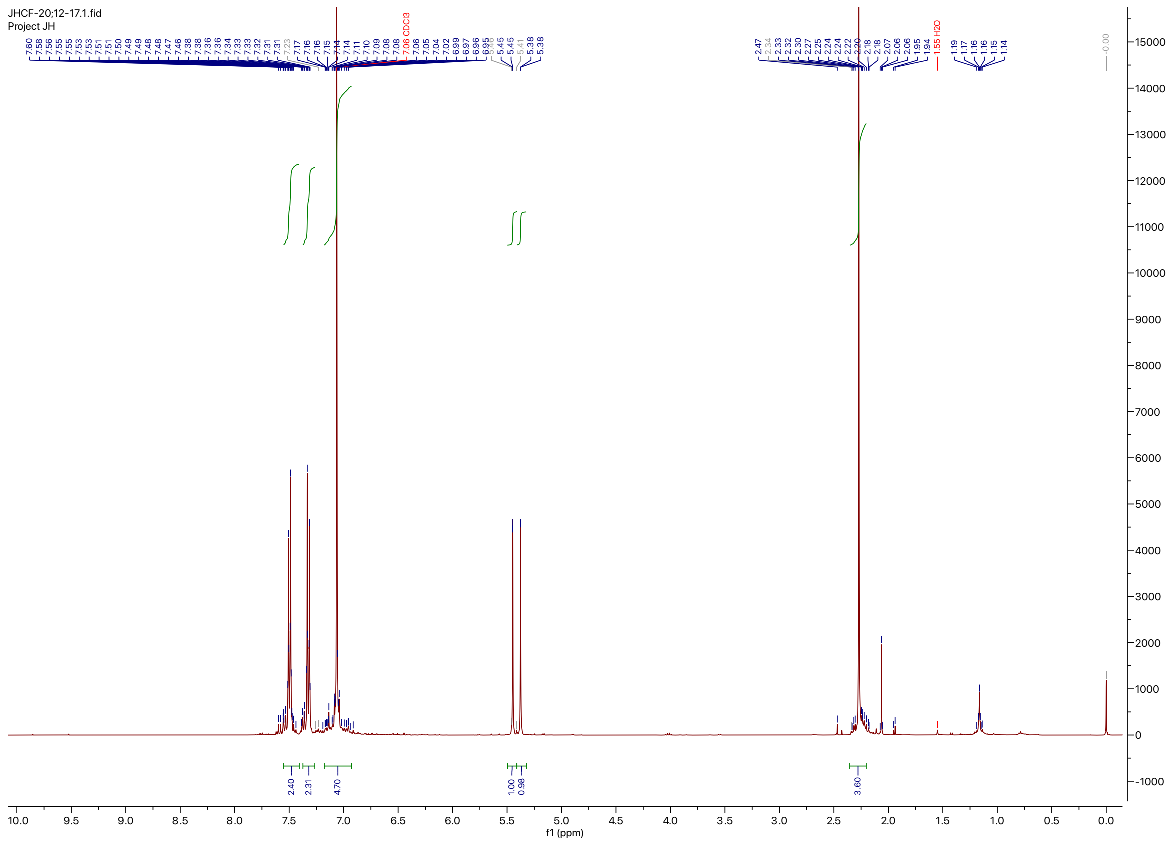

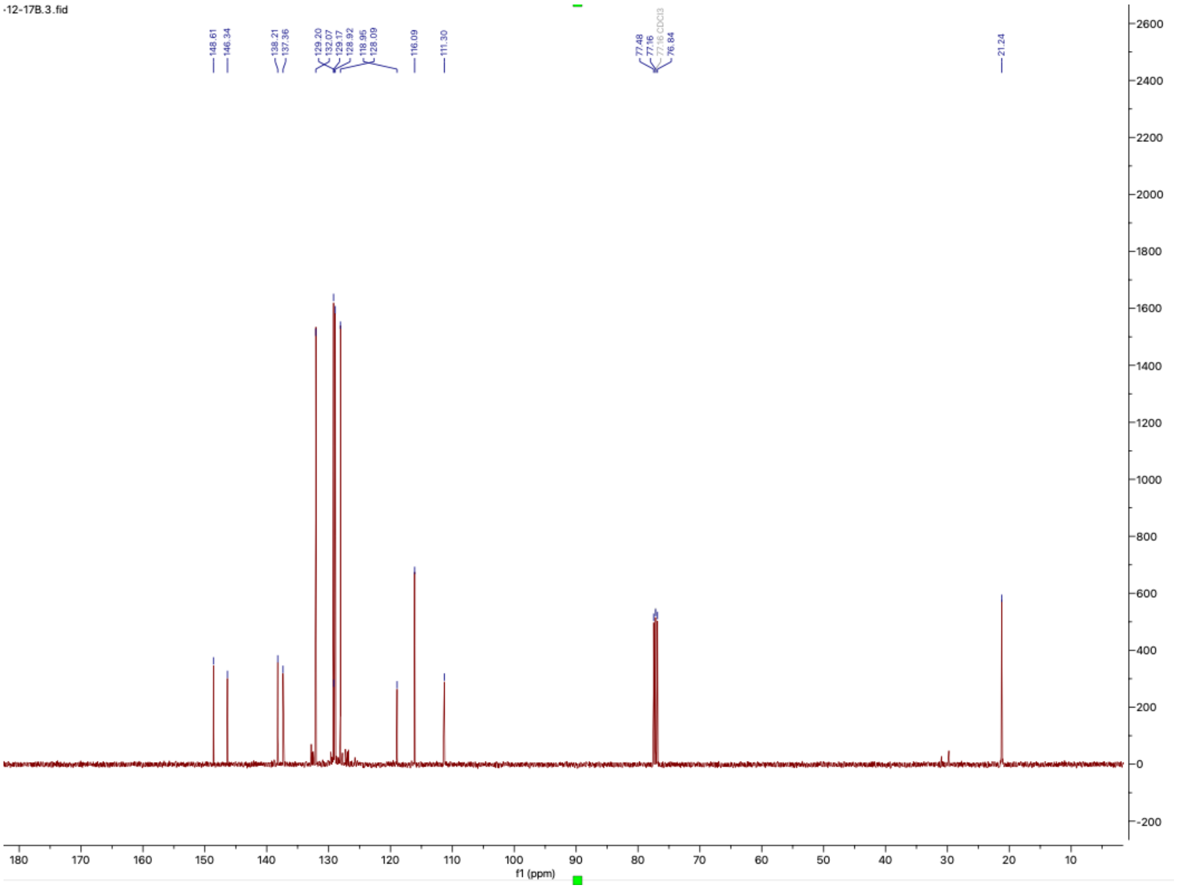

2g

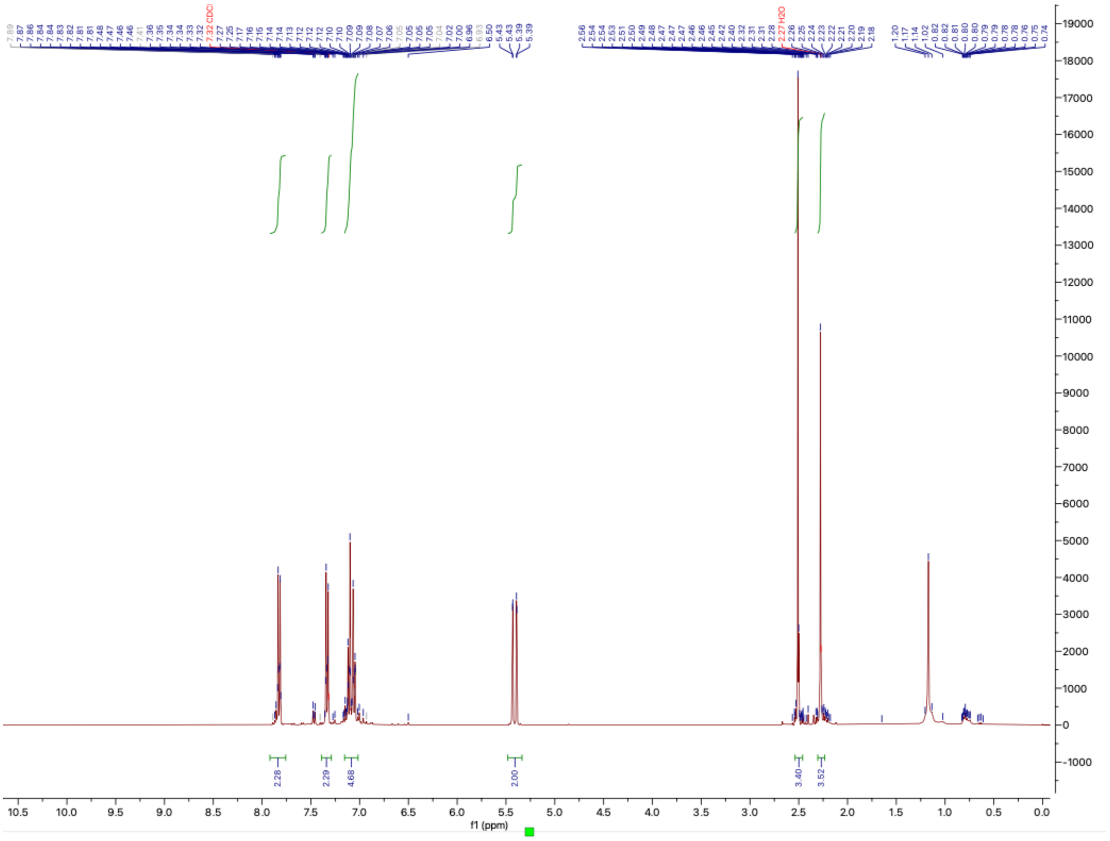

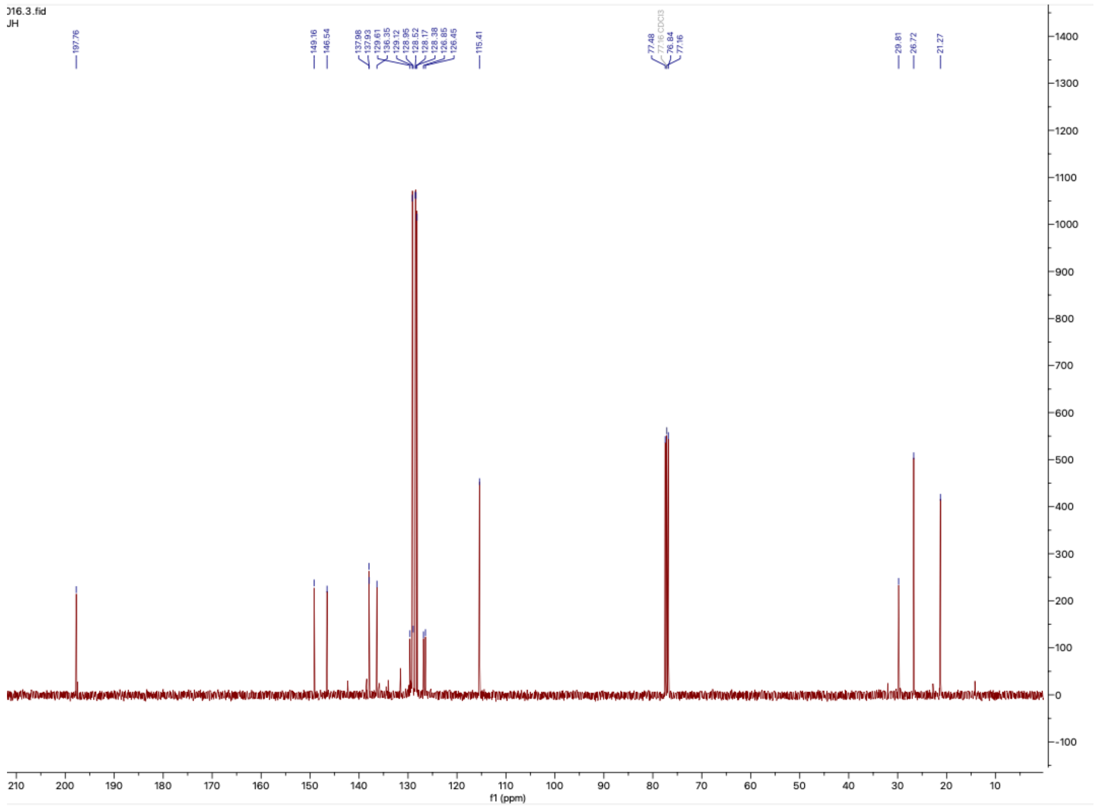

2h

^{1}H-NMR

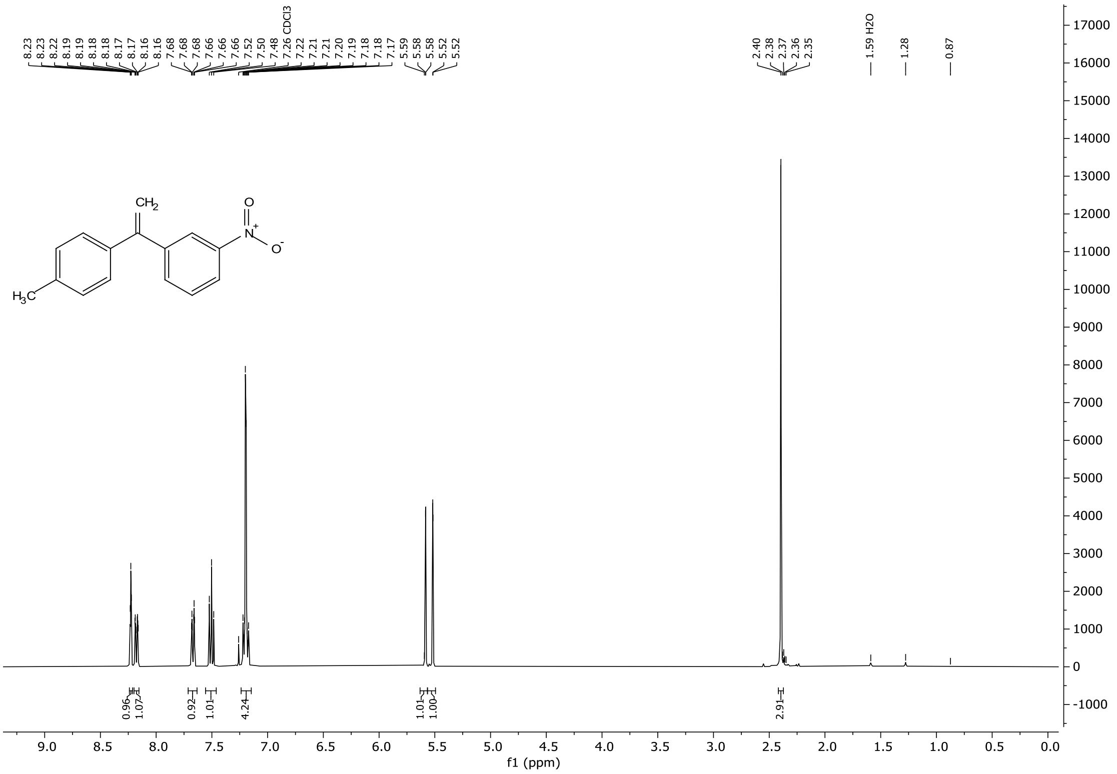

^{13}C-NMR

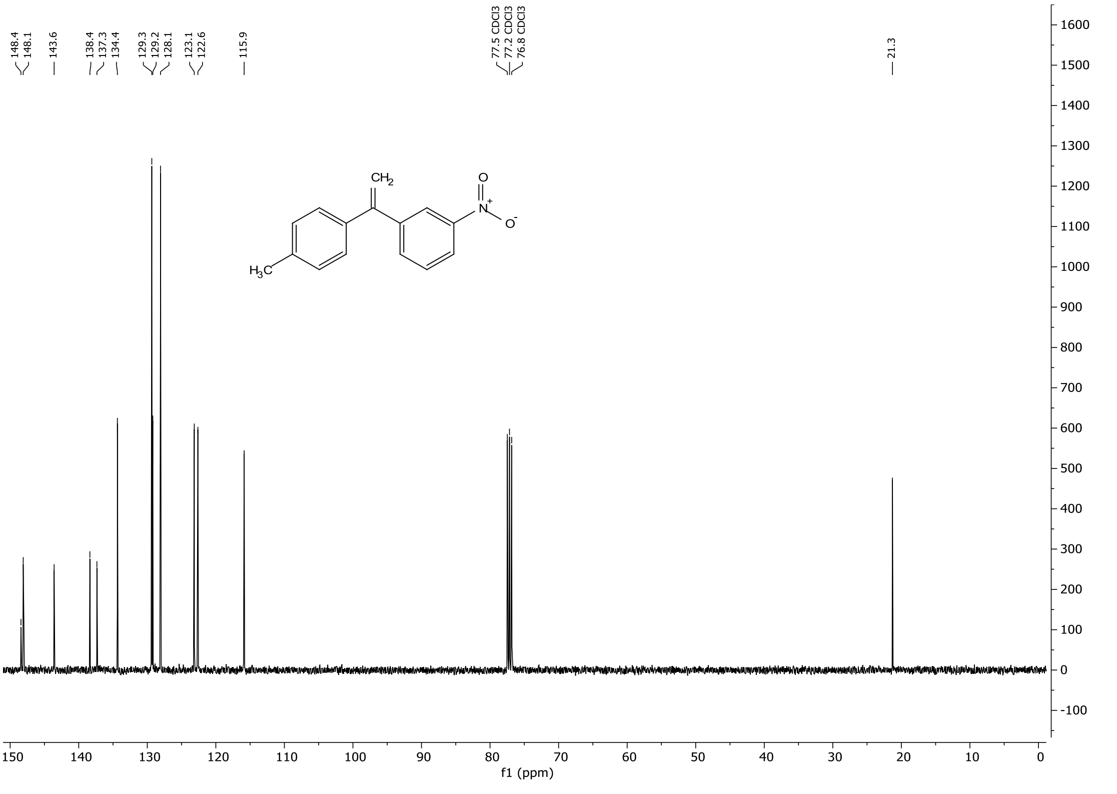

2j

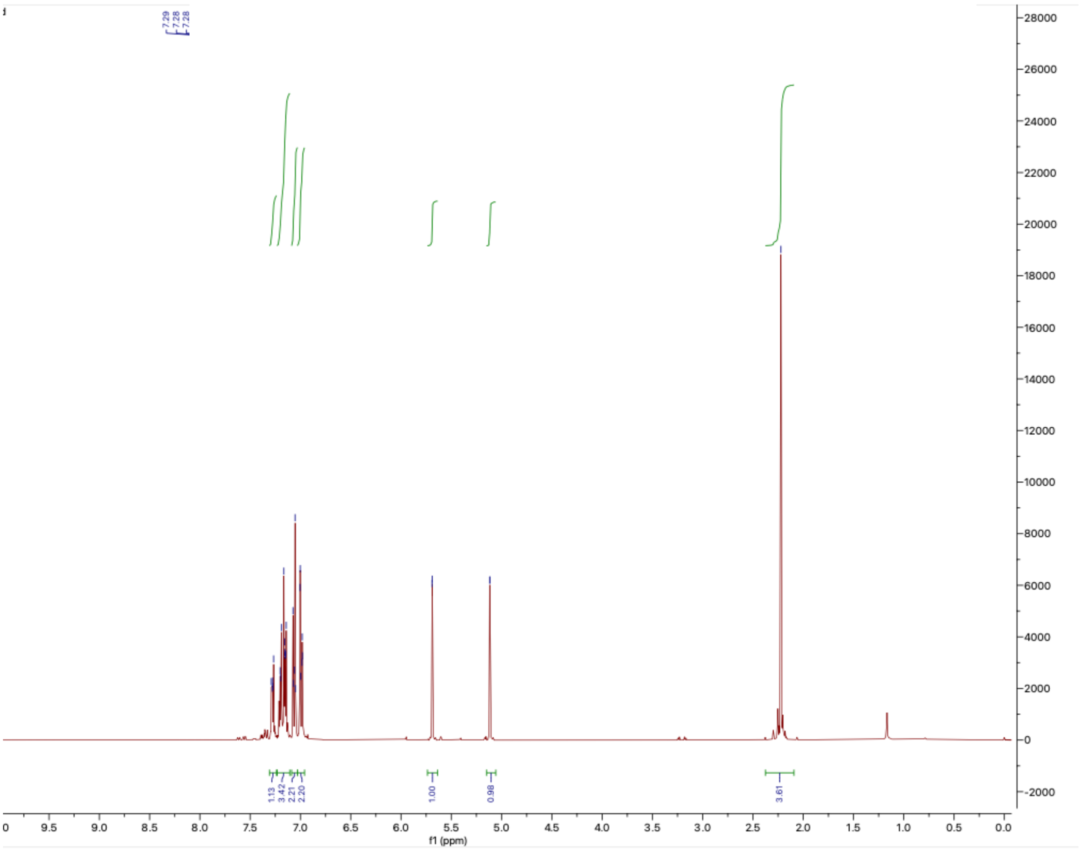
7.29
7.28
7.28
1.13
3.42
2.21
2.20
1.00
0.98
3.61
f1 (ppm)

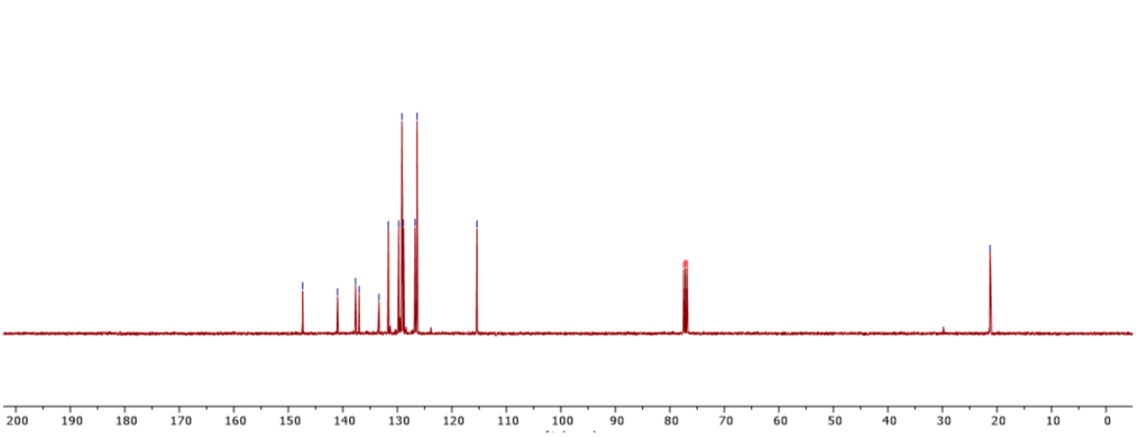
JHCF-021-FRAC3-4.3.fid
Project JH
147.37
140.98
137.66
137.01
133.38
131.67
129.77
129.16
128.86
126.75
126.39
115.39
77.46 CDCl3
77.14 CDCl3
76.83 CDCl3
21.27

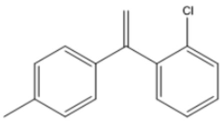
Cl

2k

^{1}H-NMR

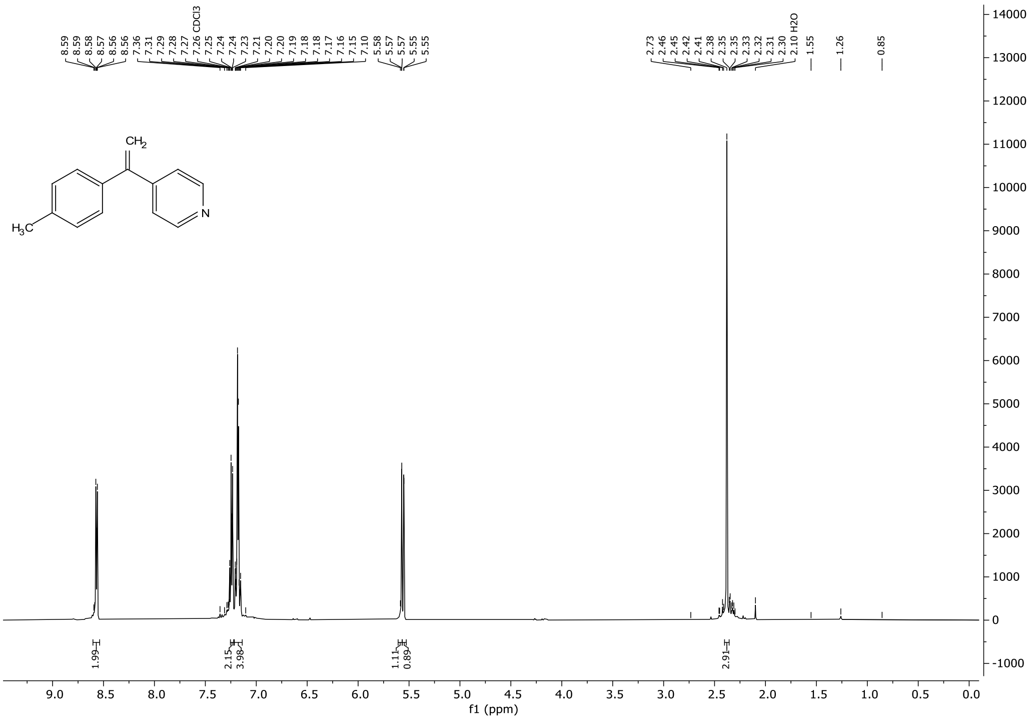

^{13}C-NMR

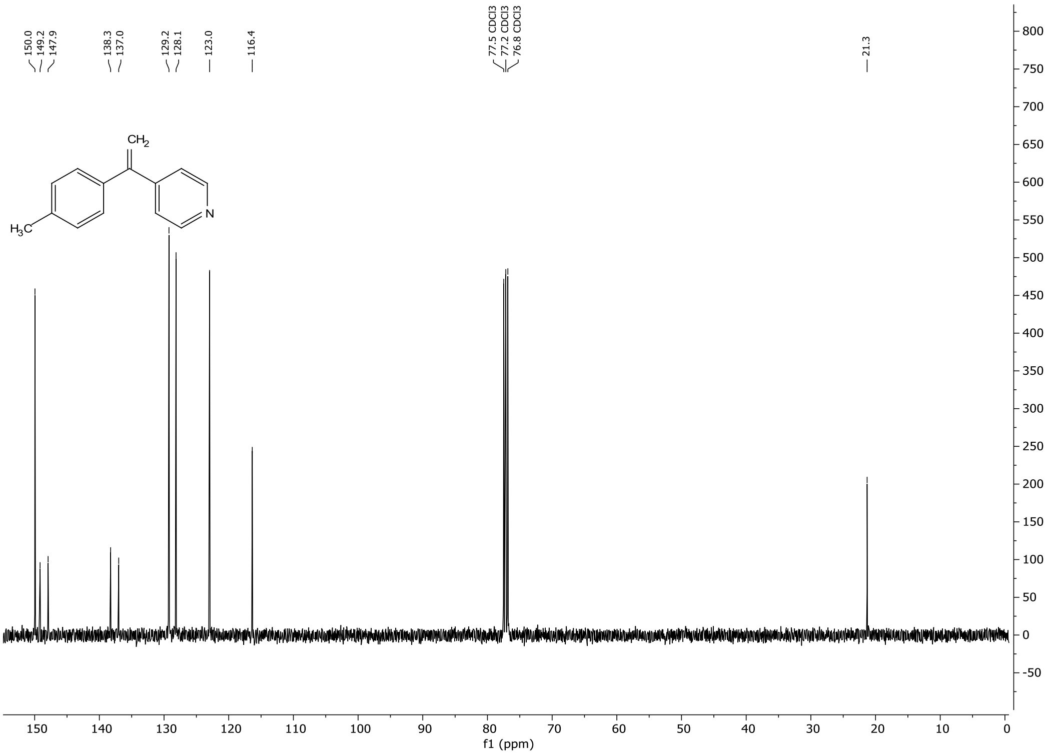

2m

^{1}H-NMR

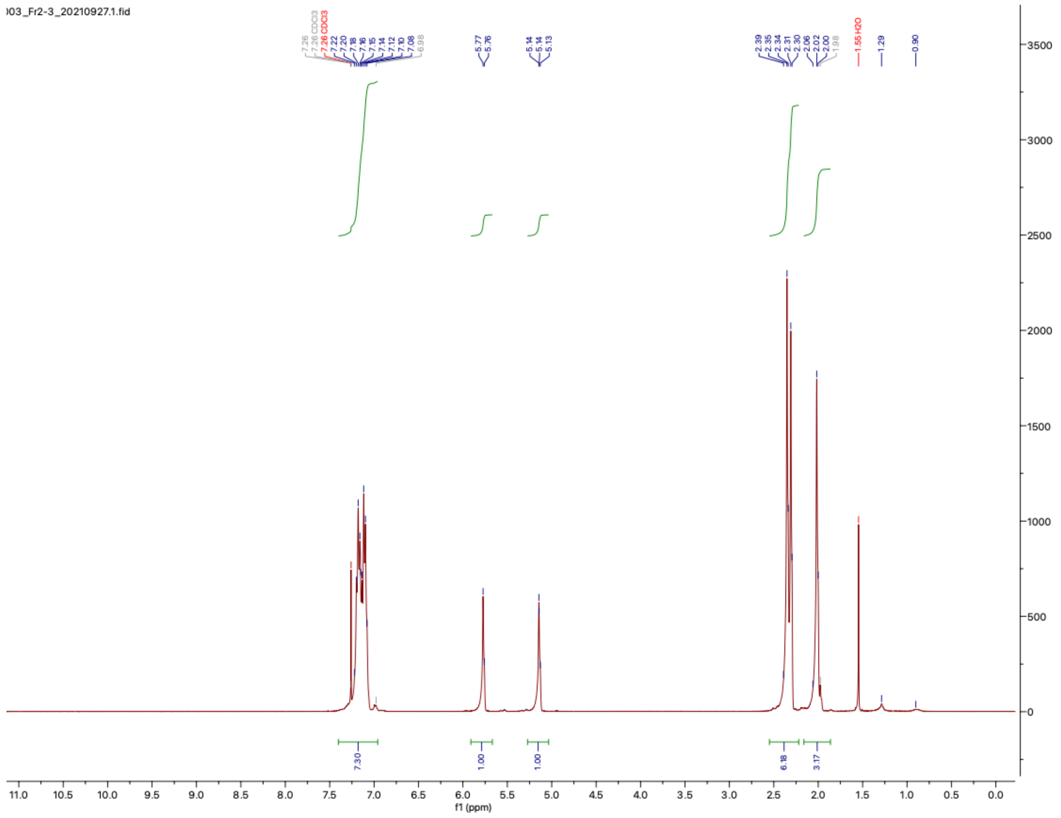

^{13}C-NMR

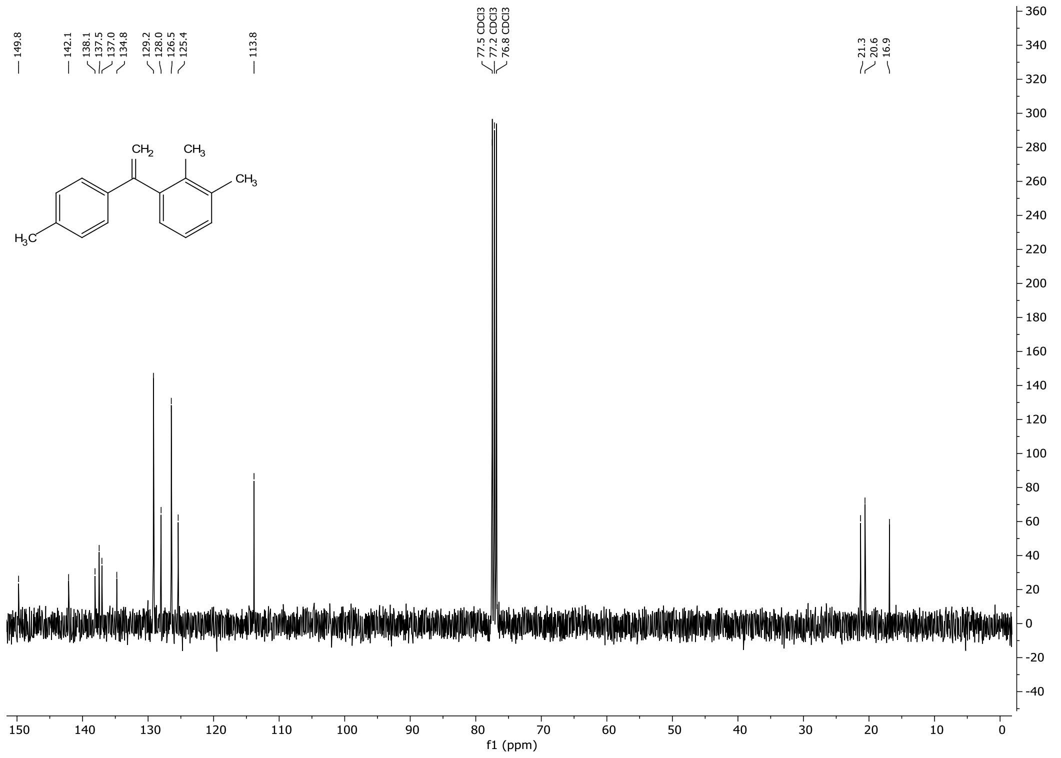

2n

^{1}H-NMR

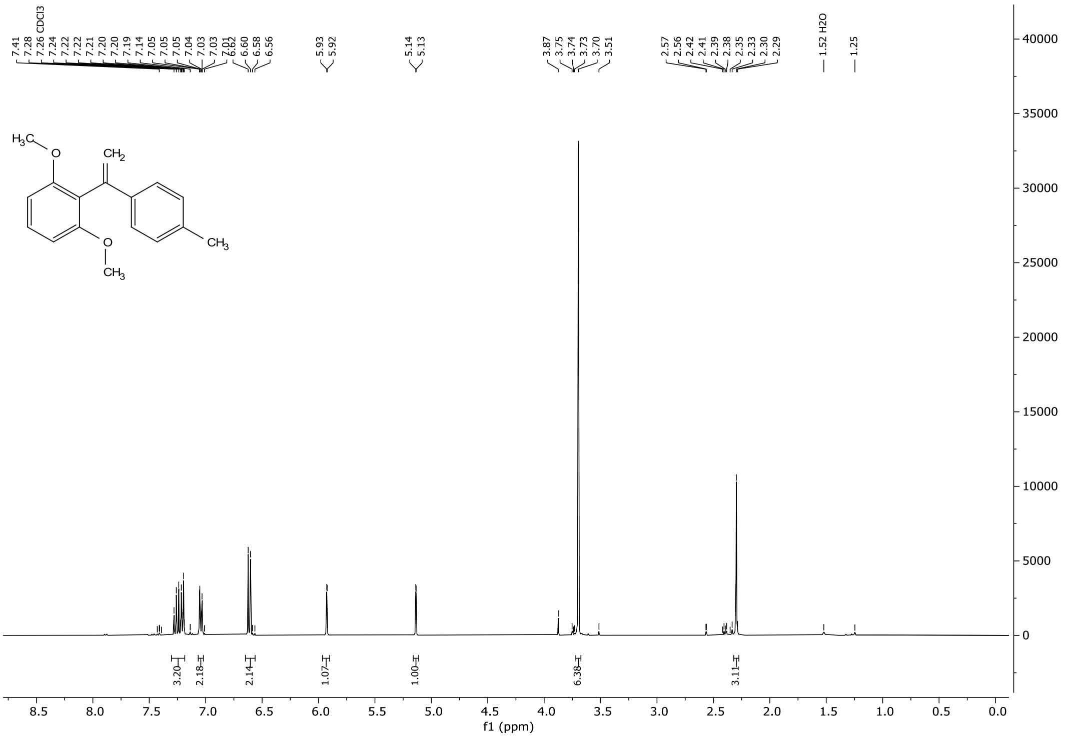

^{13}C-NMR

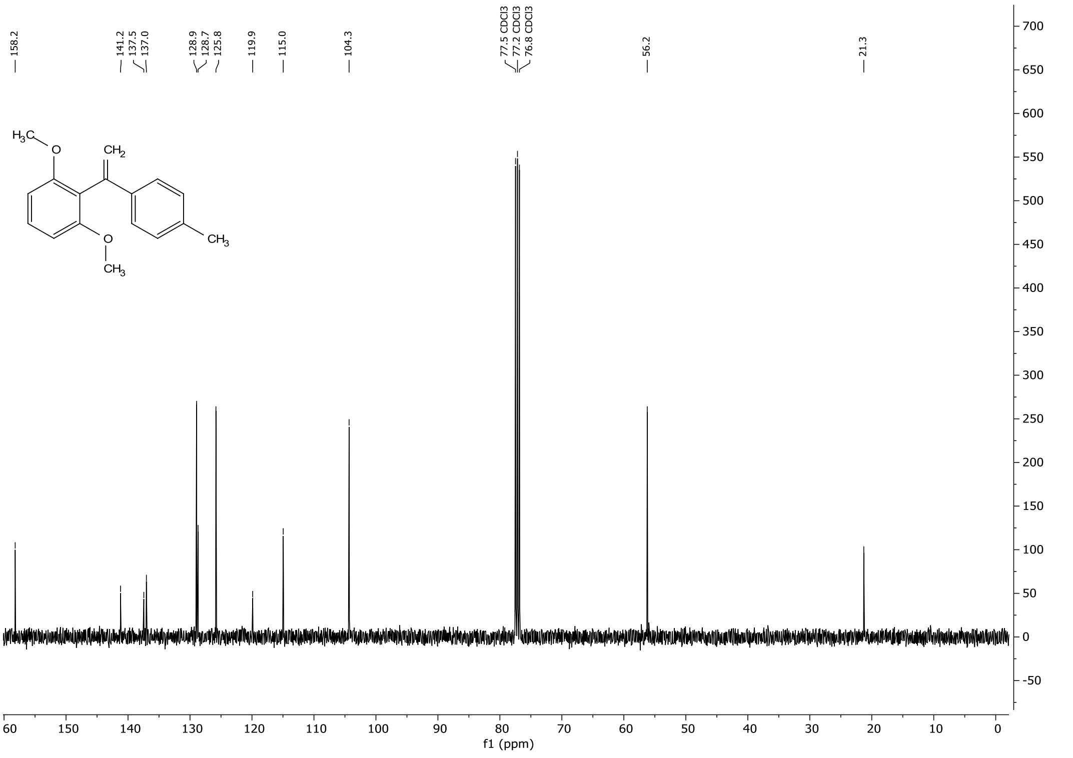

2o

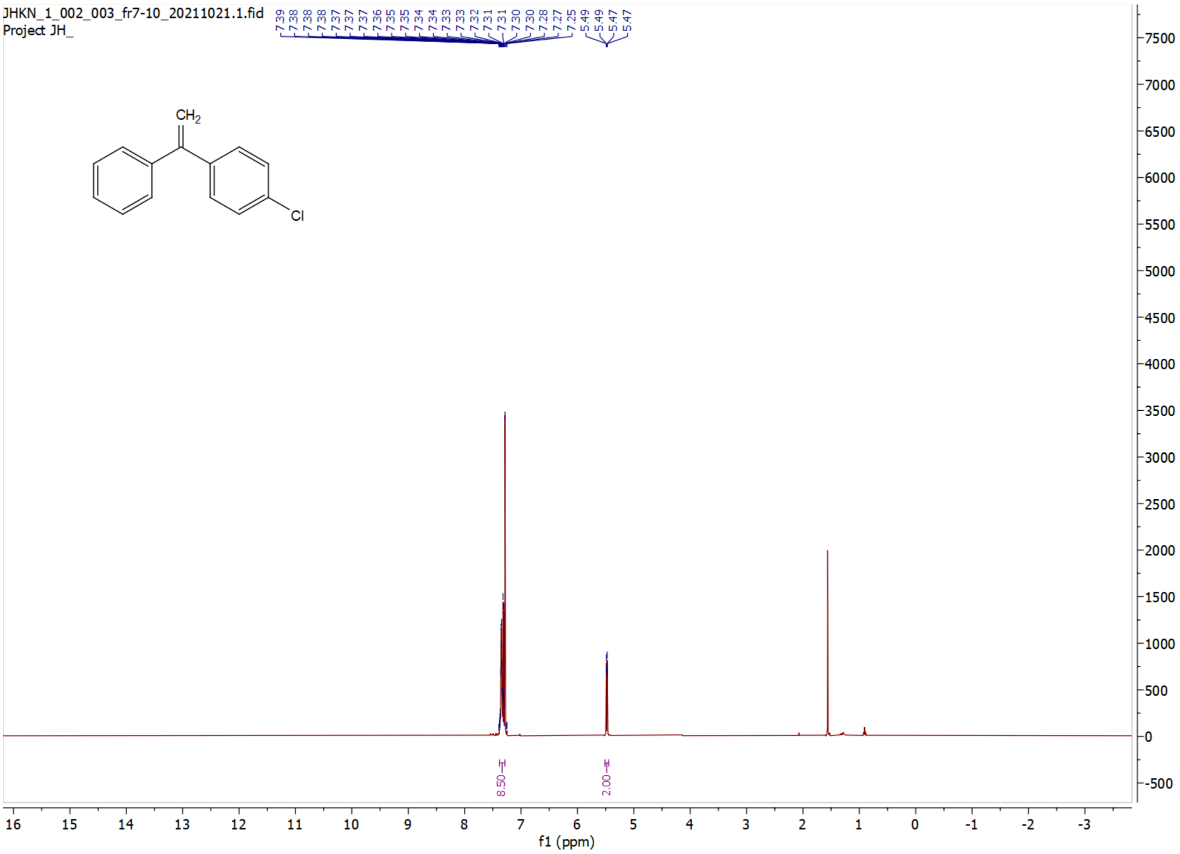
JHKN_1_002_003_fr7-10_20211021.1.fid
Project JH_
7.39
7.38
7.38
7.38
7.37
7.37
7.37
7.36
7.35
7.35
7.34
7.34
7.33
7.33
7.32
7.31
7.31
7.30
7.30
7.28
7.27
7.25
5.49
5.49
5.47
5.47
CH2
Cl
8.50
2.00
f1 (ppm)

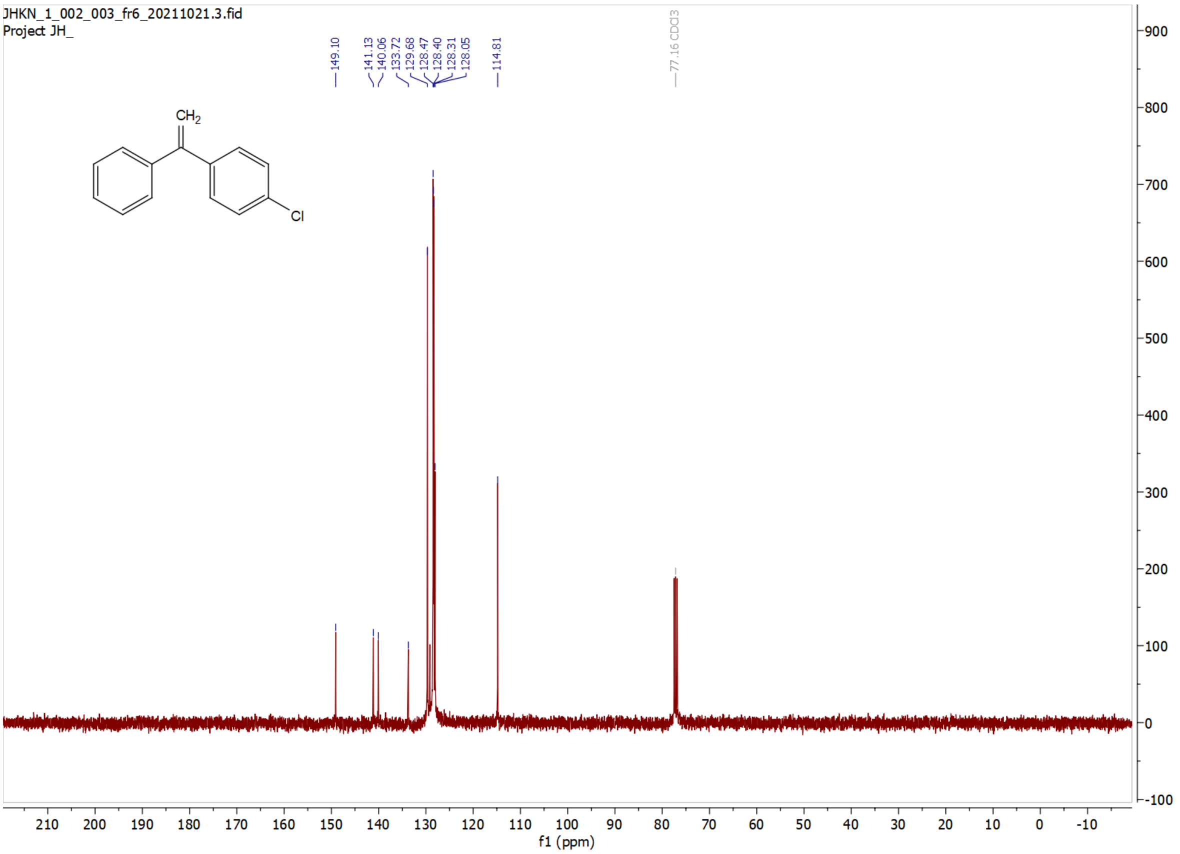
JHKN_1_002_003_fr6_20211021.3.fid
Project JH_
149.10
141.13
140.06
133.72
129.68
128.47
128.40
128.31
128.05
114.81
77.16 CDCl3
CH2
Cl
f1 (ppm)

2p

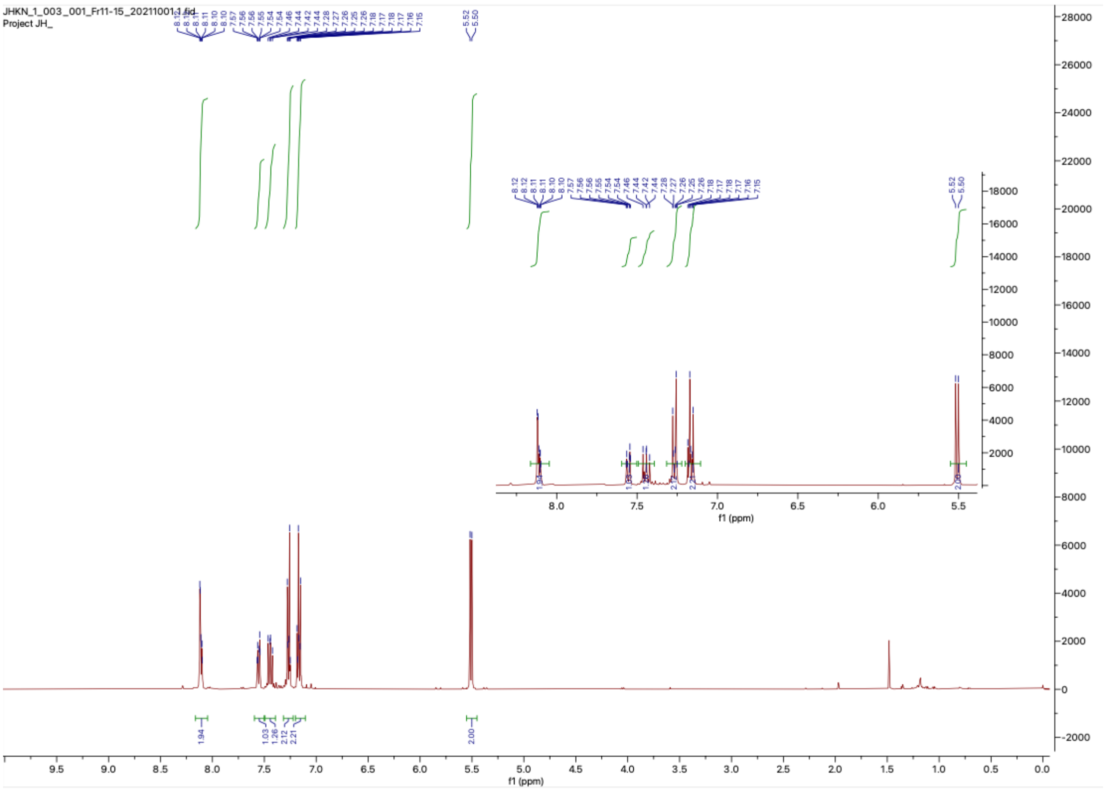
JHKN_1_003_001_Fr11-15_20211001.1.fid
Project JH_
f1 (ppm)

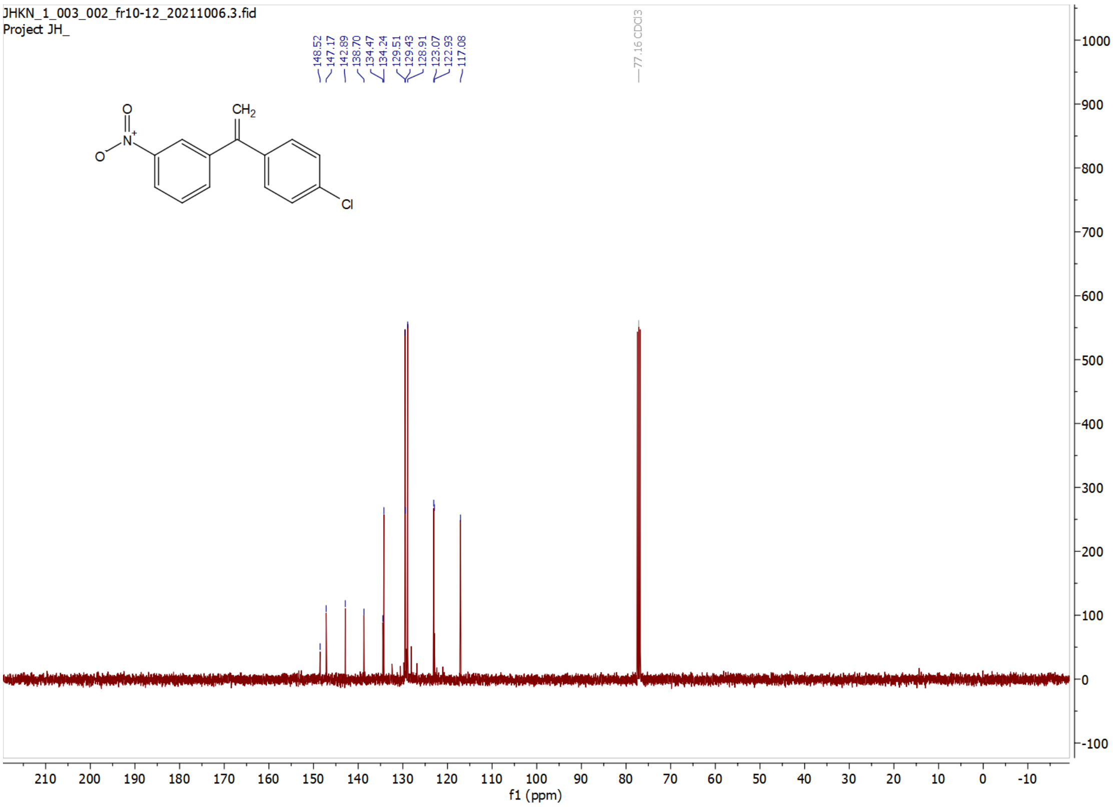
JHKN_1_003_002_fr10-12_20211006.3.fid
Project JH_
148.52
147.17
142.89
138.70
134.47
134.24
129.51
129.43
128.91
123.07
122.93
117.08
77.16 CDCl3
CH2
Cl
f1 (ppm)

2q

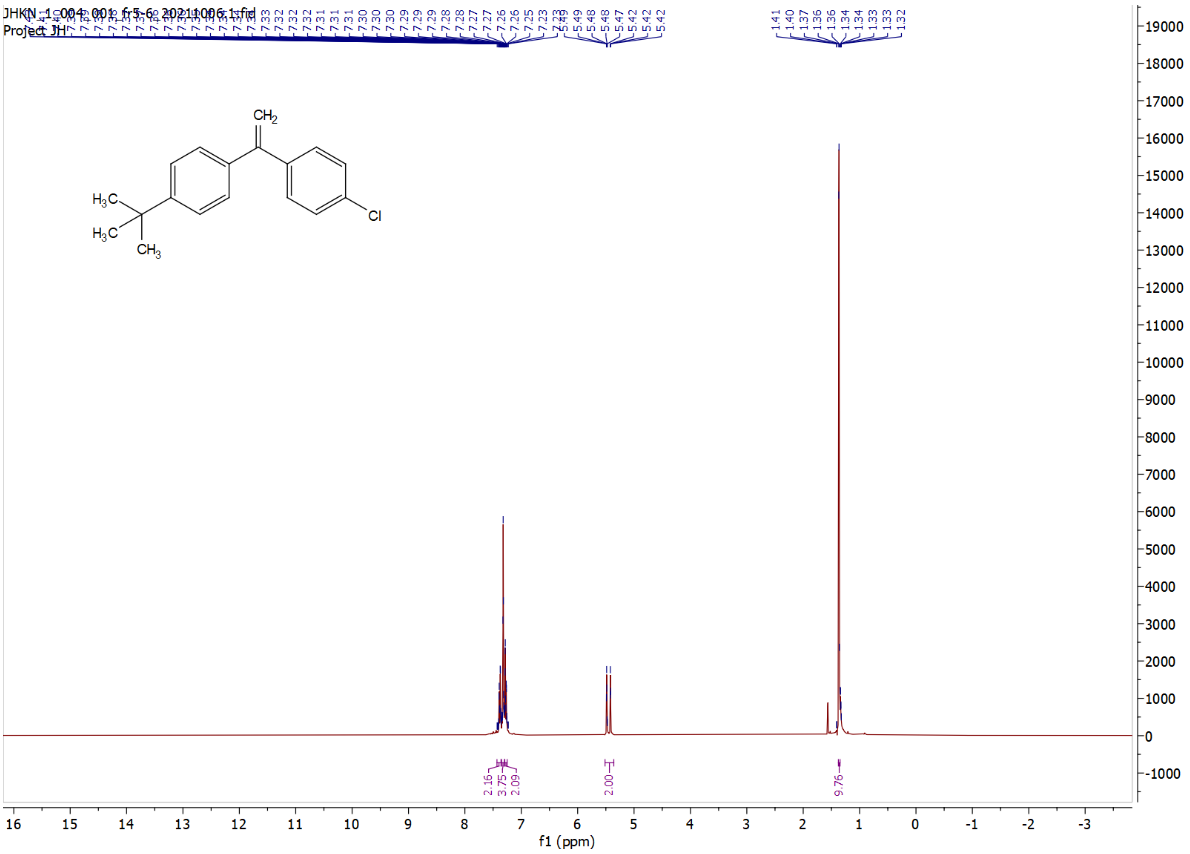

JHKN_1_004_001_fr5-6_20211006.1.fid
Project JH_
CH2
H3C
H3C
CH3
Cl
2.16
3.75
2.09
2.00
9.76
f1 (ppm)

1

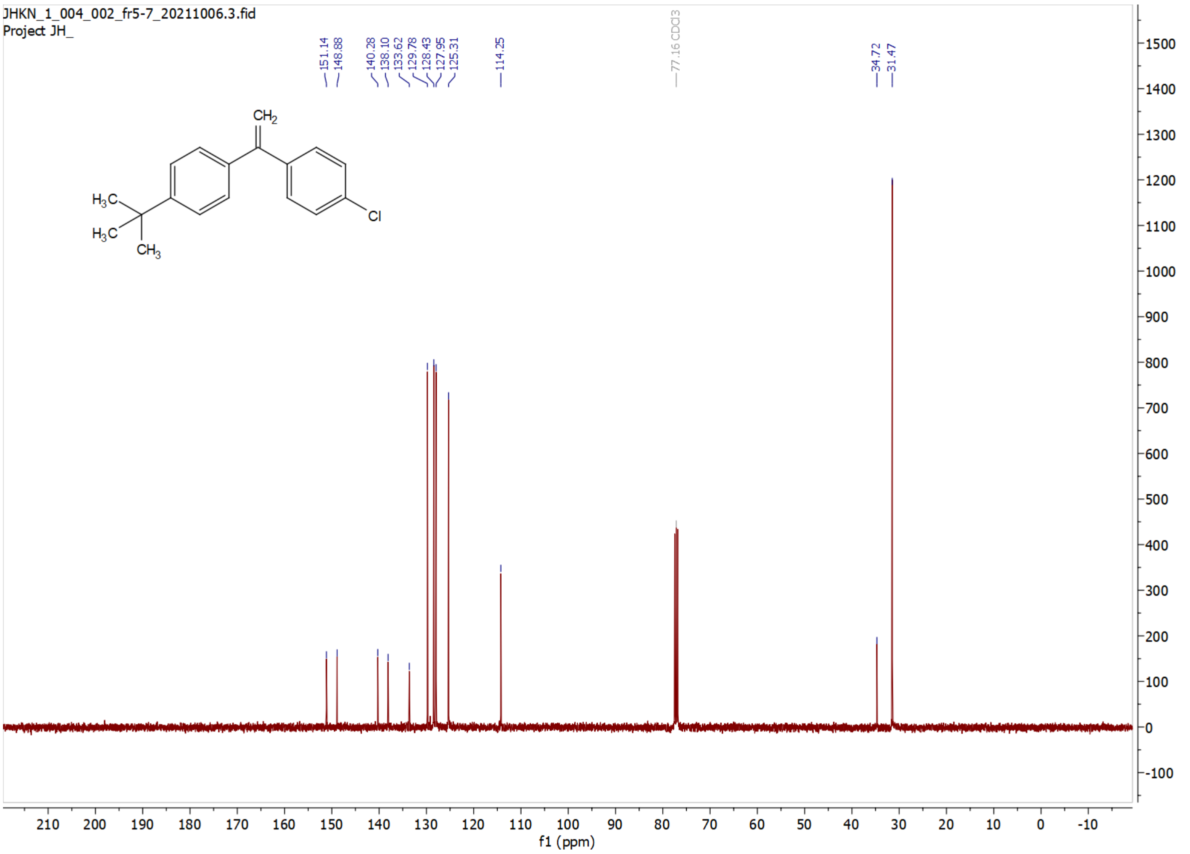

JHKN_1_004_002_fr5-7_20211006.3.fid
Project JH_
151.14
148.88
140.28
138.10
133.62
129.78
128.43
127.95
125.31
114.25
77.16 CDCl3
34.72
31.47
CH2
H3C
H3C
CH3
Cl
f1 (ppm)

References

Chen, J., Chen, C., Ji, C., & Lu, Z. (2016). Cobalt-catalyzed asymmetric hydrogenation of 1,1-diarylethenes. *Organic Letters, 18*(7), 1594–1597. https://doi.org/10.1021/acs.orglett.6b00453

Duong, U. T., Gade, A. B., Plummer, S., Gallou, F., & Handa, S. (2019). Reactivity of carbenes in aqueous nanomicelles containing palladium nanoparticles. *ACS Catalysis, 9*(12), 10963–10970. https://doi.org/10.1021/acscatal.9b04175

Gao, W.-C., Fan, J., Wei, Y.-F., Zhang, J., Chang, H.-H., & Tian, J. (2024). N-vinylthio phthalimides (N-VTPs): Modular reagents for vinylthio AIEgen transfer. *Organic Letters, 26*(1), 78–88.

Isbrandt, E. S., Chapple, D. E., Tu, N. T. P., Dimakos, V., Beardall, A. M. M., Boyle, P. D., Rowley, C. N., Blacquiere, J. M., & Newman, S. G. (2024). Controlling reactivity and selectivity in the Mizoroki-Heck reaction: High throughput evaluation of 1,5-diaza-3,7-diphosphacyclooctane ligands. *Journal of the American Chemical Society, 146*(8), 5650–5660. https://doi.org/10.1021/jacs.3c14612

Jenthra, S., Mondal, T., Kemper, G., Lantzius-Beninga, M., Hölscher, M., & Leitner, W. (2023). Ligand-controlled palladium-catalyzed decarboxylative Heck coupling for regioselective access to branched olefins. *ACS Catalysis, 13*(10), 10085–10094.

Maekawa, H., & Nishiyama, Y. (2015). Selective introduction of a trifluoroacetyl group onto 4-vinylpyridines through magnesium-promoted reduction. *Tetrahedron, 71*(35), 6694–6700.

Qin, L. N., Hirao, H. J. M., & Zhou, J. R. (2013). Regioselective Heck reaction of aliphatic olefins and aryl halides. *Chemical Communications, 49*(87), 10236–10238. https://doi.org/10.1039/c3cc45911j

Tang, J., Hackenberger, D., & Goossen, L. J. (2016). Branched arylalkenes from cinnamates: Selectivity inversion in Heck reactions by carboxylates as deciduous directing groups. *Angewandte Chemie International Edition, 55*(40), 11296–11300.

Xia, X., Chen, X., Zhao, B., & Yuan, Y. (2023). Iron-catalyzed intermolecular C-C bond vinylation of cycloketoximes promoted by diboron. *Tetrahedron, 130*, 133179.
